# Effects of butyrate^−^ on ruminal Ca^2+^ transport: evidence for the involvement of apically expressed TRPV3 and TRPV4 channels

**DOI:** 10.1007/s00424-021-02647-7

**Published:** 2022-01-31

**Authors:** Franziska Liebe, Hendrik Liebe, Gerhard Sponder, Stefan Mergler, Friederike Stumpff

**Affiliations:** 1 Institute of Veterinary Physiology, Freie Universität Berlin, Oertzenweg 19b, 14163 Berlin, Germany; 2 Department of Biology, Chemistry, and Pharmacy, Freie Universität Berlin, Berlin, Germany; 3 Institute of Experimental Ophthalmology, Charité - Universitätsmedizin Berlin, Berlin, Germany; 4 Institute of Physiology, Health and Medical University, Schiffbauergasse 14, 14467 Potsdam, Germany

**Keywords:** TRPV4, TRPV3, Rumen, SCFA, Butyric acid, GSK1016790A, GSK2193874, 2-APB, Calcium, Ammonia, Ammonium

## Abstract

The ruminal epithelium absorbs large quantities of NH_4_^+^ and Ca^2+^. A role for TRPV3 has emerged, but data on TRPV4 are lacking. Furthermore, short-chain fatty acids (SCFA) stimulate ruminal Ca^2+^ and NH_4_^+^ uptake in vivo and in vitro, but the pathway is unclear. Sequencing of the bovine homologue (bTRPV4) revealed 96.79% homology to human TRPV4. Two commercial antibodies were tested using HEK-293 cells overexpressing bTRPV4, which in ruminal protein detected a weak band at the expected ~ 100 kDa and several bands ≤ 60 kDa. Immunofluorescence imaging revealed staining of the apical membrane of the *stratum granulosum* for bTRPV3 and bTRPV4, with cytosolic staining in other layers of the ruminal epithelium. A similar expression pattern was observed in a multilayered ruminal cell culture which developed resistances of > 700 Ω · cm^2^ with expression of *zonula occludens-1* and claudin-4. In Ussing chambers, 2-APB and the TRPV4 agonist GSK1016790A stimulated the short-circuit current across native bovine ruminal epithelia. In whole-cell patch-clamp recordings on HEK-293 cells, bTRPV4 was shown to be permeable to NH_4_^+^, K^+^, and Na^+^ and highly sensitive to GSK1016790A, while effects of butyrate^−^ were insignificant. Conversely, bTRPV3 was strongly stimulated by 2-APB and by butyrate^−^ (pH 6.4 > pH 7.4), but not by GSK1016790A. Fluorescence calcium imaging experiments suggest that butyrate^−^ stimulates both bTRPV3 and bTRPV4. While expression of bTRPV4 appears to be weaker, both channels are candidates for the ruminal transport of NH_4_^+^ and Ca^2+^. Stimulation by SCFA may involve cytosolic acidification (bTRPV3) and cell swelling (bTRPV4).

**Supplementary Information:**

The online version contains supplementary material available at 10.1007/s00424-021-02647-7.

## Introduction

Arguably, the era of modern transport physiology began with Ussing’s famous discovery of active transport across amphibian skin [105]. Over 70 years later, we still know very little about transport processes across stratified squamous epithelia of mammalian species. Extraordinarily tight due to multiple layers of cells interconnected by tight junction proteins, such epithelia play a formidable role in the formation of a barrier between the external and the internal milieu of the body as in the human skin [8]. However, it is frequently overlooked that cells are also interconnected by gap junctions [65], the mutation of which induces a spectrum of hereditary diseases [10]. By facilitating exchanges of ions, molecules, and water, gap junctions interconnect cells to form a functional syncytium which in principle can mediate epithelial transport.

A classic example for a transporting cornified stratified epithelium in mammals is the ruminal epithelium of cattle and sheep [37, 93]. Interest in this tissue has historically been high since insufficient transport capacity of the rumen may lead to malnutrition and disease in ruminants [6, 63]. Apart from the suffering of the animals, inefficient use of nutrients is associated with economic losses and a negative environmental impact [22]. There is thus a direct interest in understanding more about the underlying ruminal transport processes, with potential repercussions for understanding the function of these proteins in other tissues, such as the oesophageal mucosa or the skin.

The rumen must be considered a fairly “modern” organ, with adaptation of pre-existing pathways to their current use. Triggered by the replacement of primordial forests by grasslands [94], the rumen evolved from the oesophagus about 50 million years ago [39], roughly at the same time when primates entered the stage. Within the rumen, microbes break up plant material such as grass that cannot be digested by mammalian enzymes [9]. The rumen can thus be seen as a fermentation vat to produce microbiota that are digested in the stomach and intestine, providing the ruminant with protein, fat, and vitamins. Simultaneously, fermentation products such as SCFA and ammonia (NH_4_^+^ or NH_3_) are continuously absorbed across the ruminal wall into the hepatic circulation [1, 6, 95], where they can be utilized for the synthesis of sugars, fats, non-essential amino acids, and urea. Furthermore, ruminants have perfected the ability to absorb Na^+^, Ca^2+^, and Mg^2+^ from the rumen [55, 63, 83, 118]. Despite this and due to the large quantities lost with milk, cows frequently have deficiencies in Ca^2+^ and Mg^2+^.

The mechanisms behind ruminal magnesium transport have been studied in detail. Hypomagnesemia in cattle is usually associated with an oversupply of K^+^ in conjunction with deficiencies in dietary Na^+^ and Mg^2+^ content [63, 64]. In contrast, hypocalcaemia continues to be a poorly understood although very frequent problem in dairy cattle, affecting 25–50% of the animals at the onset of lactation [24, 118]. Even if death during the acute episode can be averted by treatment, follow-up problems are severe. The rapid decrease in blood Ca^2+^ observed under parturition in cattle appears to be related primarily to the failure to mobilize sufficient Ca^2+^ from bone to compensate for the large quantities lost with milk. An important co-factor is the metabolic alkalosis of the ruminant, which interferes with parathyroid hormone activity [36]. Treatment options include lowering the cation–anion difference of the diet, thus inducing a shift in acid–base balance towards metabolic acidosis. A classical approach is adding ammonium salts to the feed [72]. As will be discussed below, ruminal absorption of ammonium occurs primarily in the form of NH_4_^+^, thus shifting protons from the rumen into blood—a process that should simultaneously stabilize systemic and ruminal acid–base balance. On the other hand, loss of ammonium from the rumen in more physiological feeding situations decreases the amount of nitrogen available for microbial protein synthesis [77]. In animals on physiological, low-protein diets, most of this nitrogen will re-enter the rumen as urea with subsequent conversion to buffering NH_3_ [1, 77]. However, in most contemporary feeding situations, large quantities of nitrogen are excreted into the environment, leading to nitrification of surface waters and formation of climate gas, both with catastrophic consequences [23, 106].

Decades of research on animals in vivo and epithelia in vitro have established that uptake of Na^+^, Ca^2+^, Mg^2+^, and ammonia from the rumen requires transcellular, protein-mediated transport pathways [55]. The ruminal uptake of Mg^2+^ involves the classical Mg^2+^ channels TRPM6 and TRPM7 [78, 85] and possibly other non-selective TRP channels [99]. Despite numerous attempts by different authors, mRNA for the classical epithelial calcium channels TRPV5 or TRPV6 could not be found in rumen of cattle or sheep [32, 78, 83, 116, 117]. Furthermore, the short-circuit current (*I*_sc_) across the epithelium is mediated by a non-selective cation channel that is very poorly sensitive to amiloride or aldosterone [62] and thus distinct from the epithelial sodium channels found in other epithelia. Functional data argue for an efflux of ammonia in the form of NH_4_^+^ via this pathway, explaining the high losses of nitrogen from the rumen even at an acidic ruminal pH [3, 11, 76, 78]. Note that when ruminal pH drops from 6.4 to 5.5, the concentration of NH_3_ drops from 0.5% of total ammonia to 0.06%. The failure to observe a corresponding decrease in ammonia absorption argues against simple diffusive efflux.

Due to its lone pair of electrons, the NH_3_ molecule is highly polar which severely limits diffusion across lipid bilayers [114]. Instead, it has emerged that transport proteins such as Rh glycoproteins or aquaporins (AQP) are required to mediate transport of NH_3_ across biological membranes [14, 34, 114]. Furthermore, both K^+^ channels [19] and K^+^ transporters such as NKCC [50] can transport NH_4_^+^, which is not surprising since the biophysical properties of both ions are similar. Functional data from in vitro studies of the ruminal epithelium show that at an unphysiologically high pH of 7.4, absorption of ammonia involves diffusion of NH_3_ [3]—possibly via AQP3 [120] since Rh glycoproteins do not appear to be expressed [119]. However, at the more physiological pH of ~ 6.4 found in vivo, uptake of NH_4_^+^ clearly predominates [3, 11, 13]. The non-selective cation channel TRPV3 has emerged as a likely candidate mediating transport not only of Na^+^ but also of NH_4_^+^ and Ca^2+^ across the ruminal epithelium [56, 78, 79], with possible participation of other channels.

Mammals express genes for at least 28 different transient receptor potential (TRP) subunits which form homomeric or heteromeric assemblies around a pore region with variable selectivity for monovalent and divalent cations [18]. The first member of this family was identified in retinae of *Drosophila* flies which showed a transient rather than a sustained receptor potential in response to light [67]. Perhaps this is why most initial research was devoted to understanding more about the involvement of TRP channels in sensory functions and signalling. Thus, TRPV3 was originally associated with thermosensation, although later studies of knockout mice and human mutations suggest a role in the cornification of the skin via pathways that have not been completely clarified [69]. In the rumen and the intestine, a role in cation transport has emerged [32, 61, 76, 78, 79]—although this certainly does not rule out other functions.

In addition to TRPV3, we have previously detected mRNA for TRPV4 in the bovine rumen. This channel is typically expressed by epithelia and has functions that range from osmosensing in the gut [45] to promoting barrier function of the skin [10]. However, detection of mRNA does not always mean that the protein is actually expressed [16] and gives no clues on the localization within a tissue. Furthermore, it is unclear if TRPV4 conducts NH_4_^+^. Accordingly, we sequenced the bovine TRPV4 (bTRPV4), overexpressed the channel in HEK-293 cells, established corresponding antibodies, and investigated the protein expression of bTRPV4 in rumen. Immunofluorescence staining was used to localize bTRPV3 and bTRPV4 in native ruminal epithelia and in a ruminal cell culture model. To test for functional expression, agonists were used on ruminal tissues in the Ussing chamber. Furthermore, we determined the conductance of bTRPV4 to NH_4_^+^. Given that studies in vivo and in vitro have shown a strong stimulatory effect of SCFA on the transport of Ca^2+^ [44, 54, 81–83, 104, 110, 117] and ammonia [12, 13] across the rumen, we finally investigated if bTRPV3, bTRPV4, or both channels are candidates for this SCFA sensitive pathway for the uptake of cations.

## Materials and methods

### Chemicals

If not stated otherwise, all chemicals were obtained from Carl Roth (Karlsruhe, Germany) or Sigma-Aldrich (Taufkirchen, Germany).

## Animal welfare

For Ussing chamber experiments, ruminal epithelium was obtained from 5 Holstein–Friesian cows that were euthanized within the context of another study in accordance with the guidelines of German legislation, with approval by the animal welfare officer of the Bundesinstitut für Risikobewertung and under the governance of the Berlin Veterinary Health Inspectorate (Landesamt für Gesundheit und Soziales Berlin, permit T 0111/20).

For immunofluorescence staining and immunoblotting, bovine ruminal epithelium was obtained from Holstein–Friesian cattle slaughtered for meat production in a commercial abattoir (Beelitz, Germany) under control of the German authorities.

## Ruminal tissue

Tissues were removed from the ventral rumen as rapidly as possible after death (< 20 min). After stripping to remove submucosal layers, the tissue was washed rigorously in Ringer solution. For Ussing chamber measurements, tissues were transported in warm (37 °C) and gassed (95% O_2_/5% CO_2_) buffer (pH 7.4, 300 mosmol · kg^−1^) which contained (in mmol · L^−1^) 70 NaCl, 40 NaGlu (sodium gluconate), 25 NaHCO_3_, 5 glucose, 5 HEPES (4-(2-hydroxyethyl)-1-piperazineethanesulfonic acid), 2.4 K_2_HPO_4_, 1.2 CaCl_2_, 1.2 MgCl_2_, and 0.4 KH_2_PO_4_, all as described previously [78]. The Ussing chamber experiments started roughly 2 h after extraction of the tissue.

For cell isolation, a 5 cm^2^ piece of stripped ruminal mucosa was washed thoroughly in phosphate-buffered saline (PBS) without Ca^2+^ and Mg^2+^, containing 4% penicillin and streptomycin (Biochrom, Berlin, Germany) and transported to the laboratory in fresh PBS at 4 °C. For protein extraction, samples were packed in aluminium foil and shock frozen in liquid nitrogen (− 80 °C). For immunofluorescence staining, tissues were stored in formaldehyde solution (Roti®-Histofix 4%).

## Ruminal cell culture

Papillae were cut from bovine ruminal tissue, repeatedly washed in PBS without Ca^2+^ and Mg^2+^ (4% penicillin and streptomycin), and processed as described previously [30, 96] and in Supplement Part A.

## Sequencing and cloning

After sequencing (GenBank: MZ028088.1), the 2613 base pairs coding sequence of the bovine homologue of *TRPV4* (*bTRPV4*) was synthetized by Thermo Fisher Scientific (Regensburg, Germany). The *bTRPV4* gene was tagged with a twin streptavidin tag (*Strep*) placed at the C-terminus followed by a sequence for yellow fluorescent protein (*YFP*). Successfully transfected cells showed yellow fluorescence (excitation peak: 514 nm; emission peak: 527 nm). For transfection of HEK-293 cells, the *bTRPV4-Strep-YFP* construct was subcloned into a pcDNA^TM^5/TO vector (p5TO, Life Technologies, Darmstadt, Germany) using the restriction sites HindIII and XbaI (Supplement, Part B). The resulting fusion protein of bTRPV4, Strep, and YFP consisted of 1144 amino acids with a calculated molecular weight of 128.55 kDa. The two tags therefore caused a size shift of ~ 30 kDa compared to the native bTRPV4 channel protein.

Cloning of the pIRES2-*Strep*-*bTRPV3*-*AcGFP*1 vector and the p5TO-*Strep*-*bTRPV3* vector was performed as described in detail in Schrapers et al. [79]. Successfully transfected pIRES2-*Strep*-*bTRPV3*-*AcGFP*1 cells could be identified by the green fluorescence (excitation peak: 395 and 475 nm; emission peak: 509 nm).

## HEK-293 cell culture and transfection

HEK-293 cells (DSMZ, Braunschweig, Germany, 2016/06/08) were cultivated under standard conditions in Dulbecco’s modified Eagle’s medium (FG0445) supplemented with 10% FBS and 1% penicillin and streptomycin (all Biochrom) [79]. For transient transfection of HEK-293 cells, PEI (polyethylenimine, linear, MW 25,000, Polysciences, Inc., Hirschberg an der Bergstrasse, Germany) was used in a calculated protocol (http://www.cytographica.com/lab/PEItransfect.html). For characterization of bTRPV4 in whole-cell patch-clamp and fluorescence calcium imaging experiments, p5TO-*bTRPV4-Strep-YFP* and control p5TO HEK-293 cells were used 1 or 2 days after transfection. For characterization of bTRPV3 in patch-clamp experiments, HEK-293 cells were transfected with the pIRES2-*Strep*-*bTRPV3*-*AcGFP*1 vector or the empty pIRES2-*AcGFP*1 vector (control) 2 days before the experiment. For calcium imaging, HEK-293 cells were transfected with the p5TO-*Strep*-*bTRPV3* or the empty p5TO vector (control) 1 or 2 days prior to experiments to prevent interference between staining of GFP and fura-2.

## Immunoblotting

HEK-293 cells or bovine ruminal tissue were prepared as described in Supplement Part C and in Liebe et al. [57].

To detect successful overexpression, a primary mouse antibody directed against the Strep tag expressed by the p5TO-*bTRPV4-Strep-YFP* vector (1:2500; Anti Strep, #34,850, Qiagen, Hilden, Germany) was used with subsequent horseradish peroxidase–conjugated secondary horse anti-mouse IgG antibody (1:1000; #7076, Cell Signaling Technology, Frankfurt, Germany).

For direct staining of bTRPV4 protein in immunoblots of HEK-293 cells and native ruminal epithelium, two commercial TRPV4 antibodies were selected after epitope screening. The first, subsequently referred to as “Thermo” (polyclonal, rabbit TRPV4 antibody, 1:500; OSR00136W, Thermo Fisher scientific), interacted with an epitope between the amino acid 300 and 400 of human TRPV4 which has 100% identity with our sequenced bovine TRPV4. The second one “ABIN” (polyclonal, rabbit TRPV4 antibody, 1:500; ABIN1049441, antibodies-online GmbH, Aachen, Germany) recognized an epitope of 20 amino acids within the internal region of human TRPV4 with stated 100% identity to cattle. For visualization, a secondary goat anti-rabbit IgG antibody conjugated to horseradish peroxidase (1:1000; #7074, Cell Signaling Technology) was applied.

## Immunofluorescence staining

All preparation steps were performed as described in detail in Liebe et al. [56]. HEK-293 cells were stained for bTRPV4 with the Thermo (1:500) or the ABIN (1:500) antibody, diluted in goat serum (5% in PBS with Ca^2+^ and Mg^2+^; PAN-Biotech GmbH, Aidenbach, Germany), and incubated overnight (4 °C). The corresponding secondary Alexa Fluor® 594–conjugated goat anti-rabbit antibody (1:1000; A-11037, Thermo Fisher scientific) was diluted in goat serum (5% in PBS) and applied for 1 h at 37 °C. To stain cell nuclei, DAPI (4′,6-diamidine-2′-phenylindole dihydrochloride, 0.2 µg/mL; Roche, Mannheim, Germany) was used. The YFP signal occupied the third channel of the confocal laser microscope.

Likewise, native ruminal tissues were stained with the primary antibodies Thermo (1:300) or ABIN (1:200) and secondary Alexa Fluor® 594–conjugated goat anti-rabbit antibody supplemented with DAPI. Additional slices of rumen were stained with Anti TRPV3 (monoclonal, mouse TRPV3 antibody, 1:1000; ABIN863127, antibodies-online GmbH) in conjunction with Anti claudin-4 (polyclonal, rabbit claudin-4 antibody, 1:1000; AB53156, Abcam, Cambridge, UK). In contrast to Liebe et al. [56], secondary antibodies were switched using Alexa Fluor® 488–conjugated goat anti-rabbit and Alexa Fluor® 594–conjugated goat anti-mouse antibodies (both 1:1000, A-11034 and A-11032, Thermo Fisher scientific).

The ruminal cell culture model that was grown in inserts was stained with ABIN (1:200) and Anti ZO-1 (monoclonal, mouse *zonula occludens-1* antibody, 1:400; 33–9100, Thermo Fisher scientific). In addition, inserts were stained with Anti TRPV3 (1:1000) in conjunction with Anti claudin-4 (1:250). As secondary antibodies, Alexa Fluor® 594 goat anti-rabbit antibody (1:1000) and Alexa Fluor® 488–conjugated goat anti-mouse antibody (1:1000; A-11029, Thermo Fisher scientific) were used, all as established in Liebe et al. [56].

All images were obtained using a confocal laser scanning microscope (LSM 710, Carl Zeiss, Jena, Germany). Secondary antibody controls were routinely performed in parallel with goat serum (5% in PBS) only and typically showed a discreet green auto fluorescence which needed to be corrected in all samples (data not shown).

## Experiments in Ussing chambers

The measurements were performed essentially as in Rosendahl et al. [78]. Small pieces of fresh ruminal mucosa were mounted in Ussing chambers, resulting in an exposure area of 3.14 cm^2^. The serosal and mucosal sides were each exposed to 15 mL buffer solution (37 °C) gassed with 95% O_2_ and 5% CO_2_. All solutions were adjusted to an osmolality of 300 mosmol · kg^−1^ with D-mannitol. In analogy to the physiological scenario, the serosal buffers were adjusted to pH 7.4 and mucosal buffers to pH 6.4. The serosal solution contained (in mmol · L^−1^): 70 NaCl, 40 NaGlu, 25 NaHCO_3_, 5 glucose, 5 KCl, 2.4 Na_2_HPO_4_, 1.2 CaCl_2_, 1.2 MgCl_2_, 0.4 NaH_2_PO_4_, and 10 mmol · L^−1^ of the buffer MOPS (3-(N-morpholino)propanesulfonic acid) [78]. In the mucosal buffer, 40 NaGlu were replaced by 25 sodium acetate, 10 sodium propionate, and 5 sodium butyrate, buffered to pH 6.4 with 10 mmol · L^−1^ MES (2-(N-morpholino) ethanesulfonic acid). The potent TRPV4 agonist GSK1016790A or the TRP channel agonist 2-APB (Supplement, Part D) were diluted in DMSO (dimethyl sulfoxide) and directly added to the mucosal bath at ≤ 1:1000. Control tissues were treated with a corresponding amount of DMSO.

One animal was used on each experimental day. After an equilibration period of ~ 20 min in open-circuit mode, the potential was clamped to 0 mV in the short-circuit mode (Mussler Scientific Instruments, Aachen, Germany). The equivalent short-circuit current (*I*_sc_) represents the negative of the current required to clamp the potential to zero. Cations transported from the apical side (mucosal) to the basolateral side (serosal) produced a positive *I*_sc_. The conductance (G_t_) was continuously determined from the potential response to a short 100 µA current pulse and varied between 5 and 21 mS · cm^−2^. Ruminal tissues with similar transepithelial G_t_ were paired in treatment groups.

## Whole-cell experiments

Whole-cell patch-clamp measurements were performed in a continuously perfused bath chamber at 23 °C [33, 56, 78, 79] (Supplement, Part E). Experimental solutions for patch-clamp experiments were modifications of established recipes [27, 109] (Supplement, Part D). Including Ca^2+^ and Mg^2+^ in the pipette and/or the bath generally resulted in higher seal rates, higher seal stability, and lower currents (Supplement, Part D).

## Intracellular calcium fluorescence imaging

Single cell calcium fluorescence imaging was performed as described in Walcher et al. [111] using an inverted microscope (Olympus BW50WI, Olympus) connected to a digital camera (XM-10, Olympus) and CellSens Dimension software (Olympus Europa Holding GmbH, Hamburg, Germany). Excitation was continuously switched between 340 (for 2.8 s) and 380 nm (for 900 ms) via cutoff filters from light provided by a LED light source (LED-Hub by Omicron, Rodgau-Dudenhofen, Germany). Emission was detected at 510 nm and used to calculate an emission fluorescence ratio (*f*_340 nm_/*f*_380 nm_) which functions as an index of relative intracellular Ca^2+^ ([Ca^2+^]_i_) levels [38].

HEK-293 cells were seeded onto coverslips and transfected as described above. After 1 or 2 days, cells were at semi-confluence (50–70%) and were loaded with fura-2 AM (1 µmol · L^−1^; PromoCell GmbH, Heidelberg, Germany) for 25–40 min (37 °C, in incubator). Cells were washed and placed in a bath chamber at 23 °C using NaCl solution at pH 6.4 (Supplement, Part D, IV). The chamber was connected via tubing to a syringe and a pumping system to allow rapid and complete changes of the bath solution. After selection of cells using the software (~ 10 min), the bath solution (Supplement, Part D, IV) was switched from NaCl 6.4 to NaBu 6.4 and back to NaCl 6.4, in 4-min intervals. For the determination of the minimum fluorescence ratio (*R*_min_), 13 mmol · L^−1^ EGTA replaced MgCl_2_ and CaCl_2_ in the NaCl 6.4 solution. For the determination of the maximum fluorescence ratio (*R*_max_), NaCl 6.4 solution with 20 mmol · L^−1^ CaCl_2_ (but no MgCl_2_ or EGTA) was applied. The [Ca^2+^]_i_ was calculated from the emission ratio (*f*_340 nm_/*f*_380 nm_) according to Grynkiewicz et al. [38] using Igor Pro Software.

## Data analysis and statistical analysis

Data evaluation was performed using Igor Pro 6.37 (WaveMetrics Inc., Lake Oswego, USA) using the equations in Supplement Part F.

Statistical evaluation was carried out with SigmaPlot 11.0 (Systat Software, Erkrath, Germany). Within the same group, data were tested using the Friedman repeated measures analysis of variance on ranks test, followed by the Student–Newman–Keuls method. Comparisons of several groups were performed using the Kruskal–Wallis one-way analysis of variance (ANOVA) on ranks. Comparison of two groups occurred via the Mann–Whitney rank sum test. Significance of differences was assumed when *p* ≤ 0.05. Values were given as means ± standard error of the mean (SEM), rounding as recommended by the DIN 1333 [25]. The *N*-value represents the number of individual cattle in Ussing chamber experiments or, in the case of calcium imaging, the number of coverslips. The *n*-value represents the number of individual samples.

## Results

### Sequencing and overexpression of bTRPV4

The bovine homologue of *TRPV4* (*bTRPV4*) was sequenced from ruminal tissue (GenBank: MZ028088.1, encoding for the protein QXI66840.1). HEK-293 cells were transfected with the *bTRPV4-Strep-YFP* construct in a p5TO vector (Supplement, Part B).

### Immunoblot: bTRPV4 HEK-293 cells

**Fig. 1:**
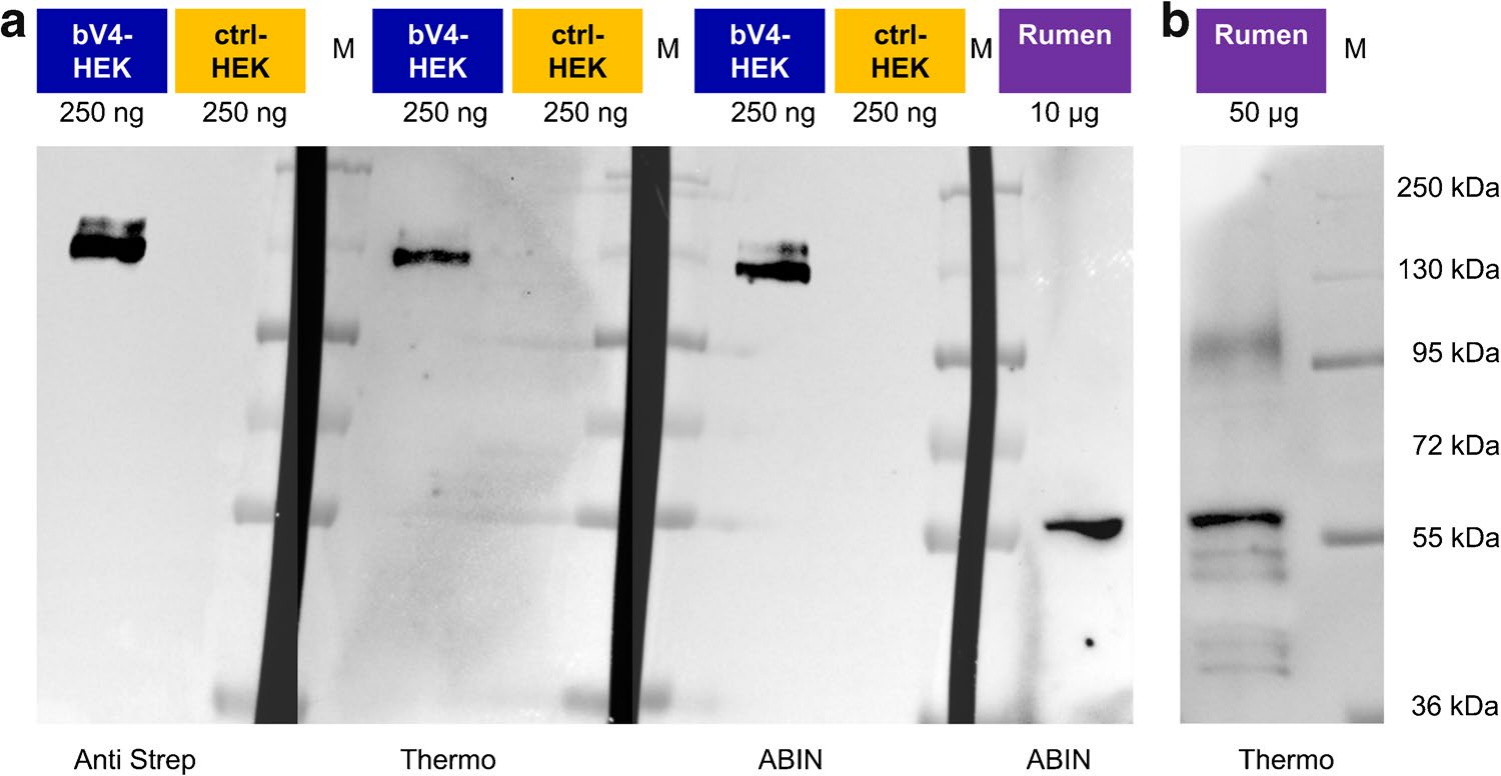
Immunoblots: detection of bTRPV4 in overexpressing HEK-293 cells and in bovine rumen. Immunoblots using the Anti Strep, ABIN, and Thermo antibodies as indicated at the bottom. Lane titles and the corresponding amount of total protein are indicated at the top. **a** Three lanes each of protein from Strep tagged bTRPV4 HEK-293 cells (bV4-HEK), control HEK-293 cells (ctrl-HEK), and marker protein (M) were blotted onto one membrane, followed by a lane with protein from the bovine rumen (rumen). This membrane was cut along the marker lanes (M, black gaps) and incubated separately using different primary antibodies. Each membrane piece was processed independently to gain ideal exposure times. Afterwards, images were gamma corrected and merged. **b** At a higher concentration of 50 µg protein from bovine rumen (rumen), a band was visible at the expected height of bTRPV4 (~ 100 kDa). Image was gamma corrected

Two antibodies directed against TRPV4 (Thermo and ABIN) both stained bTRPV4 protein from overexpressing HEK-293 cells at a similar height of ~ 130 kDa (Fig. 1a, *n* = 4). This height reflects the sum of the predicted molecular weight of bTRPV4 (~ 100 kDa) and the Strep and YFP tags. The doubling of the band most likely reflects glycosylation, as reported for TRPV4 from mouse oesophageal epithelium [87]. Bands of similar height appeared after staining against the Strep tag (*n* = 4). No band was visible in the lanes with protein of empty p5TO transfected control HEK-293 cells (*n* = 4).

### Immunoblot: native bovine ruminal epithelium

Both TRPV4 antibodies were used for detection in protein samples of the native bovine rumen (Fig. 1a, b). A strong ~ 60 kDa band could be observed using either Thermo (*n*/*N* = 12/5) or ABIN (*n*/*N* = 5/3). At high concentrations and longer exposure times, a weaker band occurred at a molecular weight of ~ 100 kDa, corresponding to the predicted native bTRPV4 sequence (Fig. 1b). The ~ 60 kDa band could again be observed, along with a number of signals with a lower molecular weight, possibly reflecting breakdown products.

### Confocal laser microscopy: HEK-293 cells

**Fig. 2:**
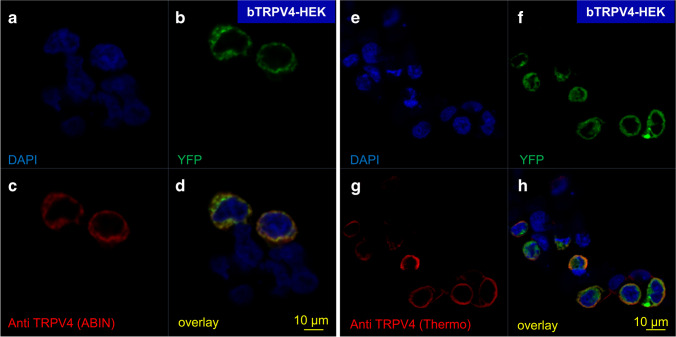
Confocal laser microscopy: localization of bTRPV4 in bTRPV4 HEK-293 cells. Cell nuclei were stained with DAPI (blue) (**a**, **e**). The YFP signal (green) indicates successful transfection with expression of the fusion protein bTRPV4-Strep-YFP (**b**, **f**). The ABIN (**c**) and the Thermo antibody (**g**) selectively stained bTRPV4 (red). Overlay with co-localization of staining for bTRPV4 and YFP is shown in yellow, primarily in the cell membrane **(d**, **h**). Cells not successfully transfected only stained for DAPI

HEK-293 cells successfully overexpressing bTRPV4 showed fluorescence for YFP, which was fused to the bTRPV4 protein and thus appeared in the cell membrane (Fig. 2b, f, green). Similarly, staining of the membrane with the ABIN (Fig. 2c) or Thermo (Fig. 2g) antibodies was observed (each *n* = 2). Since the transfection rate of PEI is estimated to be ~ 30%, a number of cells in the same cell dish did not express the bTRPV4-YFP fusion protein (Fig. 2d, h). These cells and controls exclusively showed DAPI staining.

### Confocal laser microscopy: bovine ruminal epithelium

**Fig. 3:**
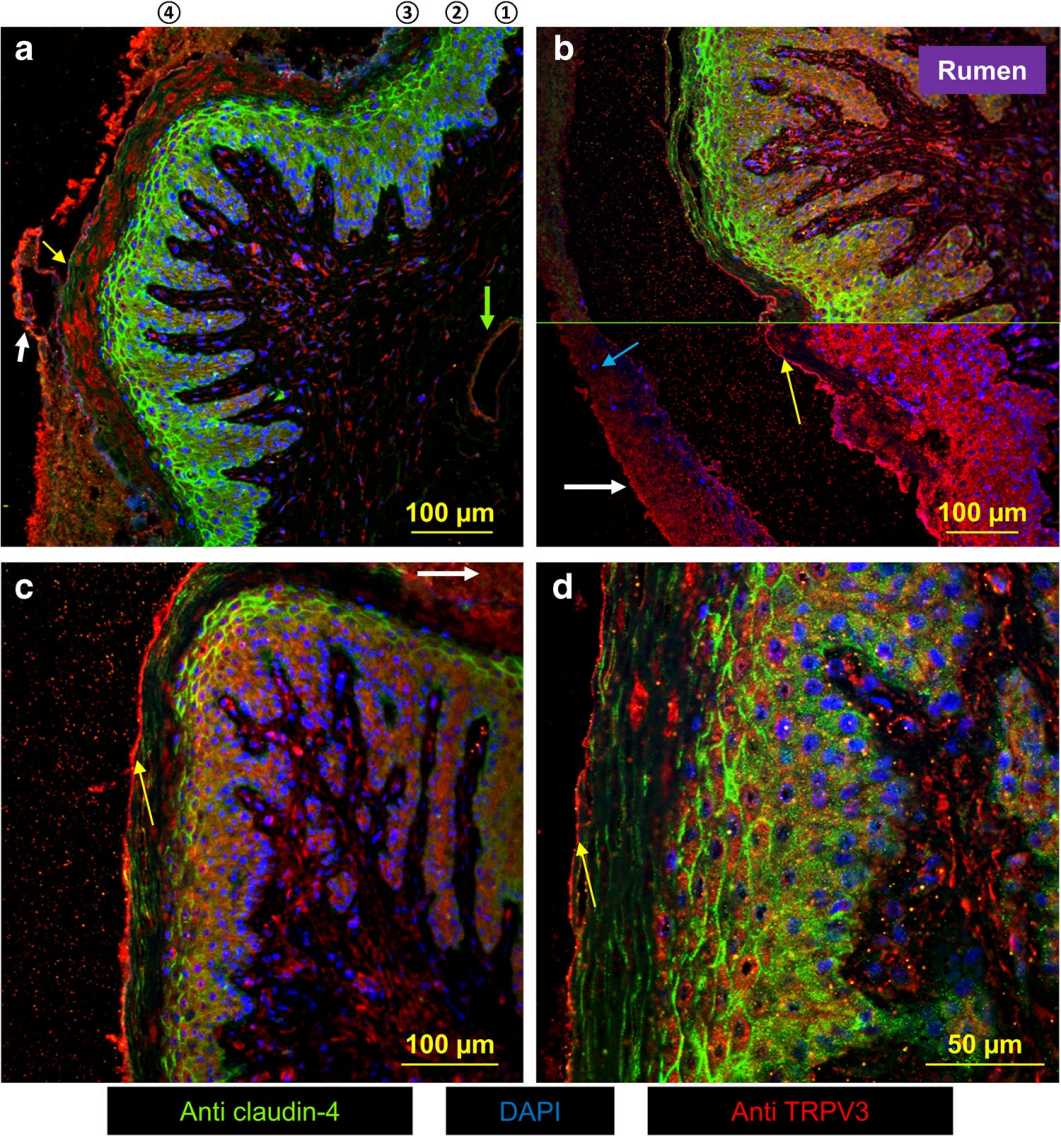
Confocal laser microscopy: localization of bTRPV3 and claudin-4 in native ruminal epithelium. bTRPV3 was stained in red (Anti TRPV3), claudin-4 in green (Anti claudin-4), and cell nuclei in blue (DAPI). **a** Staining for claudin-4 can be seen weakly in the cytosol of the *stratum basale* (①), shifting into the cell membrane in the *stratum spinosum* (②), while delicately demarking the flat cells of *stratum granulosum* (③). Note the line of bTRPV3 staining in the apical membrane (yellow arrow). Above this line, bTRPV3 staining is dysmorphic in the *stratum corneum* (④). The *stratum corneum* has detached partially (white arrow). In the subepithelial space, a blood vessel shows pronounced bTRPV3 staining (green arrow). **b** Staining for claudin-4 is only shown in the top part of the figure above the green line. The *stratum corneum* (white arrow) has detached completely, with residual cell nuclei (blue arrow) that are typical signs of parakeratosis. The point of detachment is again lined by intense bTRPV3 staining (yellow arrow). In the *stratum spinosum* and *basale*, staining for bTRPV3 is cytosolic. **c** Ruminal papillae with *stratum corneum* that has detached almost completely, except at the top right (white arrow). Note the claudin-4–stained cell boundaries in the *stratum granulosum* and the bright apical staining for bTRPV3 (yellow arrow). **d** The higher magnification shows the apical membrane of the *stratum granulosum* with intense staining for bTRPV3 (yellow arrow). Staining for claudin-4 can be seen in the cell boundaries of the *stratum granulosum* and *spinosum*. The cytosol of the *stratum granulosum* shows very little staining for bTPRV3

Bovine rumen was obtained from slaughterhouse cattle and showed a typical structure with the *stratum basale* (①), *stratum spinosum* (②), and *stratum granulosum* (③) (Fig. 3a). The *stratum corneum* (④) was partially or totally detached (Fig. 3a, b, white arrows) and contained residual cell nuclei (Fig. 3b, blue arrow). These are signs of parakeratosis, a condition associated with high-grain finishing diets [5, 53, 92, 97].

Tissues were first stained for bTRPV3 in conjunction with the tight junction protein claudin-4. Localization was essentially as reported previously [56, 96] (Fig. 3, *n*/*N* = 3/1). Weak claudin-4 staining could be seen in the cytosol of the *stratum basale*, increasingly shifting to cell boundaries in the *stratum spinosum*. Cells of the *stratum granulosum* were flat but showed clear membrane-bound staining for claudin-4 (Fig. 3). Conversely, the *stratum corneum* does not express functional tight junction proteins [8, 97]. The ubiquitous faint staining of this layer for claudin-4 and bTRPV3 without demarcation of cell boundaries almost certainly reflects dysfunctional membrane proteins dislodged by the corneocyte envelope (see 31).

All three basal layers of the ruminal epithelium (①, ②, ③) showed cytosolic staining for bTRPV3 which most likely reflects processing of bTRPV3 in the endoplasmic reticulum or expression by cell organelles. Any overlay with claudin-4 was very discrete. The apical membrane of the *stratum granulosum* appeared as a distinct red line reflecting accumulated expression of bTRPV3 (Fig. 3, yellow arrows). Apart from the strong signal in the apical membrane, staining for bTRPV3 was poor in the *stratum granulosum*. This suggests that the channel protein is mainly produced and assembled in the underlying cell layers and trafficked to the apical membrane of the functional syncytium.

**Fig. 4:**
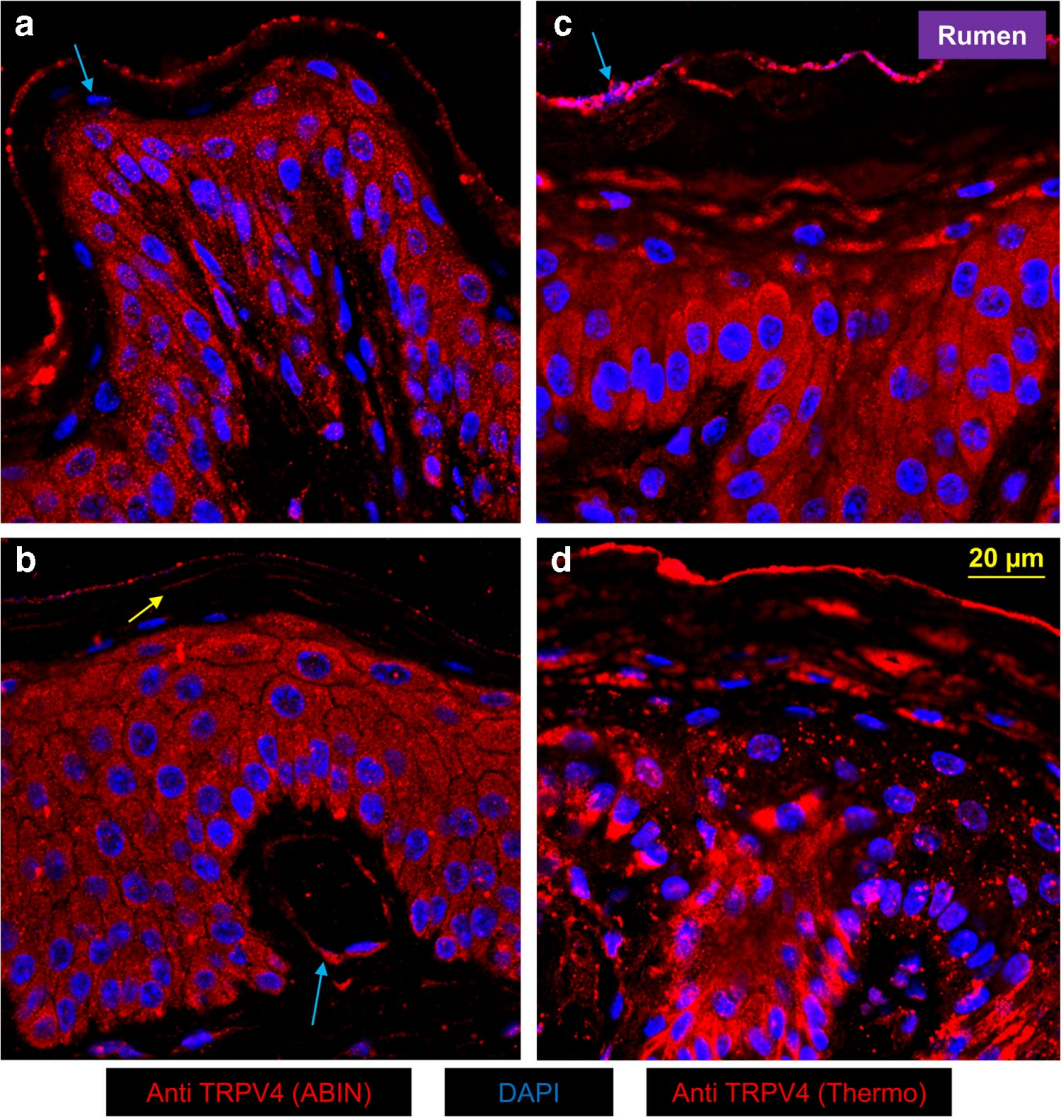
Confocal laser microscopy: localization of bTRPV4 in native ruminal epithelium. bTRPV4 was stained in red using ABIN (**a**, **b**) or Thermo (**c**, **d**), and cell nuclei were stained in blue using DAPI. Despite some variability, the cytosol tended to be intensely stained for bTRPV4 in the *stratum spinosum* and *stratum basale*. Towards the top layers, staining intensity decreased strongly. Note the small strips of bTRPV4 staining (**b**, yellow arrow). At the apical membrane of the *stratum granulosum*, an intense line of bTRPV4 staining is visible with rudimentary cell nuclei (**c**, blue arrow). Subepithelial staining was very discrete, except around blood vessels (**b**, blue arrow)

A similar staining pattern was observed when using the antibodies for bTRPV4, with cytosolic staining visible in the epithelial layers and intense staining of the apical membrane belonging to the *stratum granulosum* (Fig. 4, *n* = 38/4).

In comparison to the epithelium, the staining of the subepithelium was weak. However, pronounced staining was visible around blood vessels (Fig. 3a and 4b), indicating bTRPV3 and bTRPV4 expression by the vascular endothelium as described previously [91, 98].

### Multilayered ruminal cell culture

Ruminal keratinocytes from the bovine rumen could be cultured in inserts to form multilayered model epithelia expressing tight junction proteins, as shown in a previous study using material from sheep [96]. Interestingly, feeder cells were not needed. Four inserts with keratinocytes from 2 cows were produced. Three inserts developed transepithelial electrical resistance (TEER) values of 750, 741, and 705 Ω · cm^2^ on day 20, after which they were stained. The fourth filter dropped from 510 Ω ∙ cm^2^ on day 20 to 270 Ω ∙ cm^2^ on day 24, after which it was also stained.

**Fig. 5:**
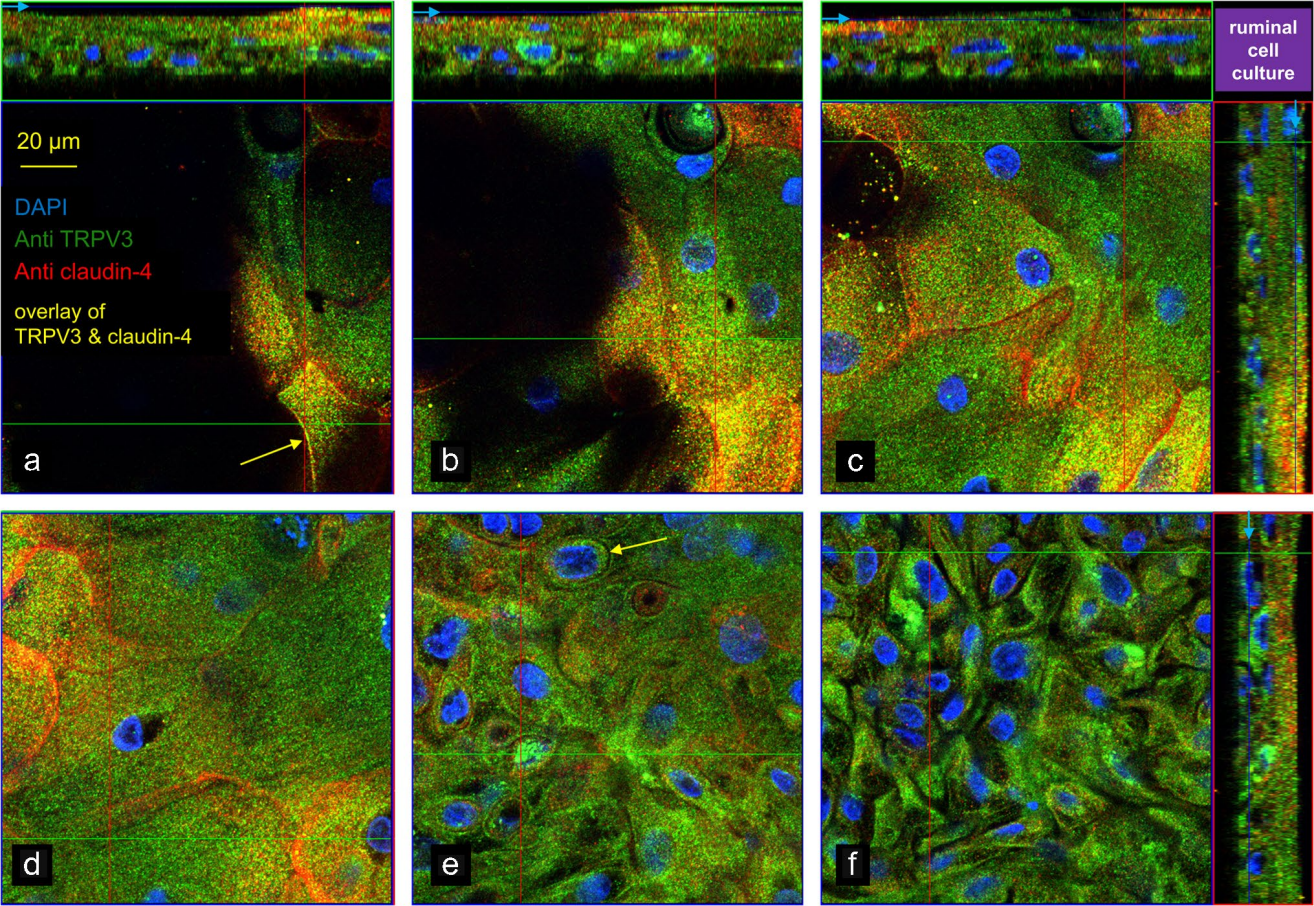
Confocal laser microscopy: localization of bTRPV3 and claudin-4 in a multilayered ruminal cell culture. Immunofluorescence staining shows bTRPV3 in green using ABIN, claudin-4 in red using Anti claudin-4 with co-expression of both proteins resulting in a yellow staining, and cell nuclei in blue using DAPI (*n*/*N* = 3/2). At the top and at the right-hand side, z-stack panels can be seen. The blue line and arrows indicate the position at which the section plane in the main panel was taken in **a**, **b**, **c**, and **f**. **a** The view of the top layer of cells showed cytosolic staining for both bTRPV3 and claudin-4, with considerable overlay. In places, co-staining is clearly visible in the cell membrane (yellow arrow). **b**, **c**, and **d** These images were taken at different points below panel **a**. Note the predominant claudin-4 staining of the cell boundaries, suggesting that membrane expression of bTRPV3 is weak. **e** A step further down, membrane expression of bTRPV3 is visible in some places (yellow arrow), becoming stronger in the cytosol, while claudin-4 is only visible in the cytosol. **f** In the bottom layer of cells, any staining for claudin-4 is rudimentary, while the cytosol continues to show staining for bTRPV3. For more detail, see the film in the supplement

**Fig. 6:**
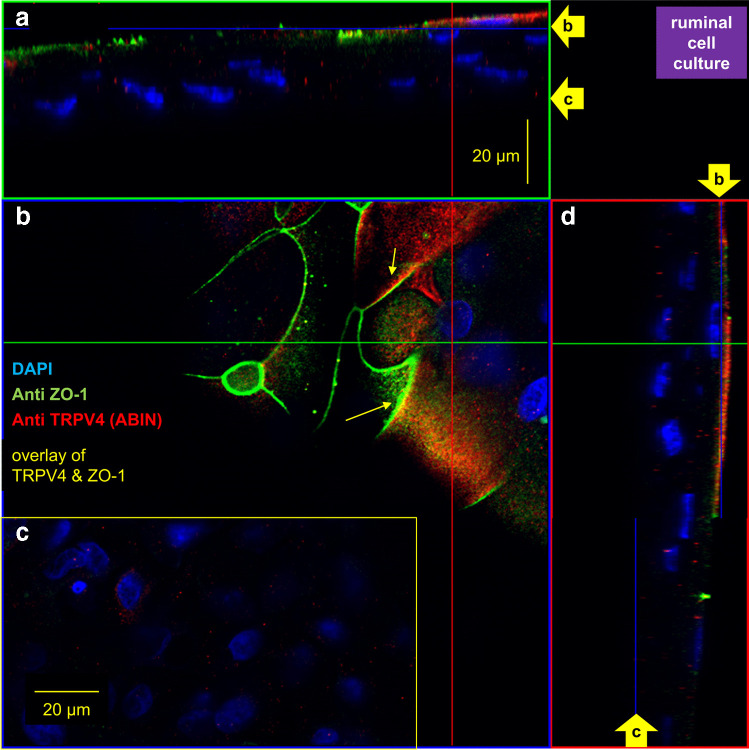
Confocal laser microscopy: localization of bTRPV4 and ZO-1 in multilayered ruminal cell culture. Immunofluorescence staining shows ZO-1 in green using Anti ZO-1, bTRPV4 in red using ABIN with co-expression of both proteins resulting in a yellow staining, and cell nuclei in blue using DAPI (*n*/*N* = 5/2). Similar images were obtained using the Thermo antibody (not shown, *n*/*N* = 2/1). **a** Cross-section through the preparation (z-stack). The filter is at the bottom of the image, and cells in this location only show staining for cell nuclei. In the top layers, additional staining for bTRPV4 and ZO-1 emerges, with little sign of co-expression. The arrows “b” and “c” at the side indicate the locations of the section planes (**b**, **c**). **b** View of a slice through the top layer. A number of cells show intense staining for ZO-1, associated with the cell membrane. At the top right, membrane staining for bTRPV4 can be seen. In a few places, discrete co-expression of bTRPV4 and ZO-1 is visible (yellow arrows). The green line shows the localization of the cross-section seen in **a**. **c** View of a slice through the bottom layer. Rudimentary cytosolic staining for bTRPV4 can be seen around the blue cell nuclei. **d** Cross-section along the red line in **a** and **b**. Again, arrows indicate the position of panels **b** and **c**. For more detail, see the film in the supplement

All filters showed staining for bTRPV3 and bTRPV4 (Fig. 5, 6, and supplemental films). Expression of ZO-1, claudin-4, and bTRPV4 was rudimentary in the basal layers near the filter (Fig. 5f, 6c), in contrast to the strong cytosolic staining for bTRPV3 (Fig. 5e, f), possibly reflecting endoplasmic processing prior to trafficking. As cells grew upwards, they began expressing claudin-4 (Fig. 5c, d), ZO-1, and bTRPV4 (Fig. 6a, d) within the cytosol. More apically, the tight junction proteins claudin-4 and ZO-1 began to stain the cell boundaries until in the most apical layers, membrane-bound staining for bTRPV3 (Fig. 5a) and bTRPV4 (Fig. 6b) became visible, all in striking resemblance to what was found in the native epithelium. However, in cell culture, there were signs of a co-localization of bTRPV3 and claudin-4 as well as of bTRPV4 and ZO-1.

## Functional studies of bovine ruminal epithelium in the Ussing chamber

To screen for functional expression of bTRPV4 in native tissues, Ussing chamber experiments were performed using the specific TPRV4 agonist GSK1016790A [7, 107] in ruminal tissues of 5 cows. To ensure vitality of the tissues, we tested effects of 2-APB in parallel. This is a classical agonist which activates a number of TRP channels including TRPV3, but not TRPV4 [46].

**Fig. 7:**
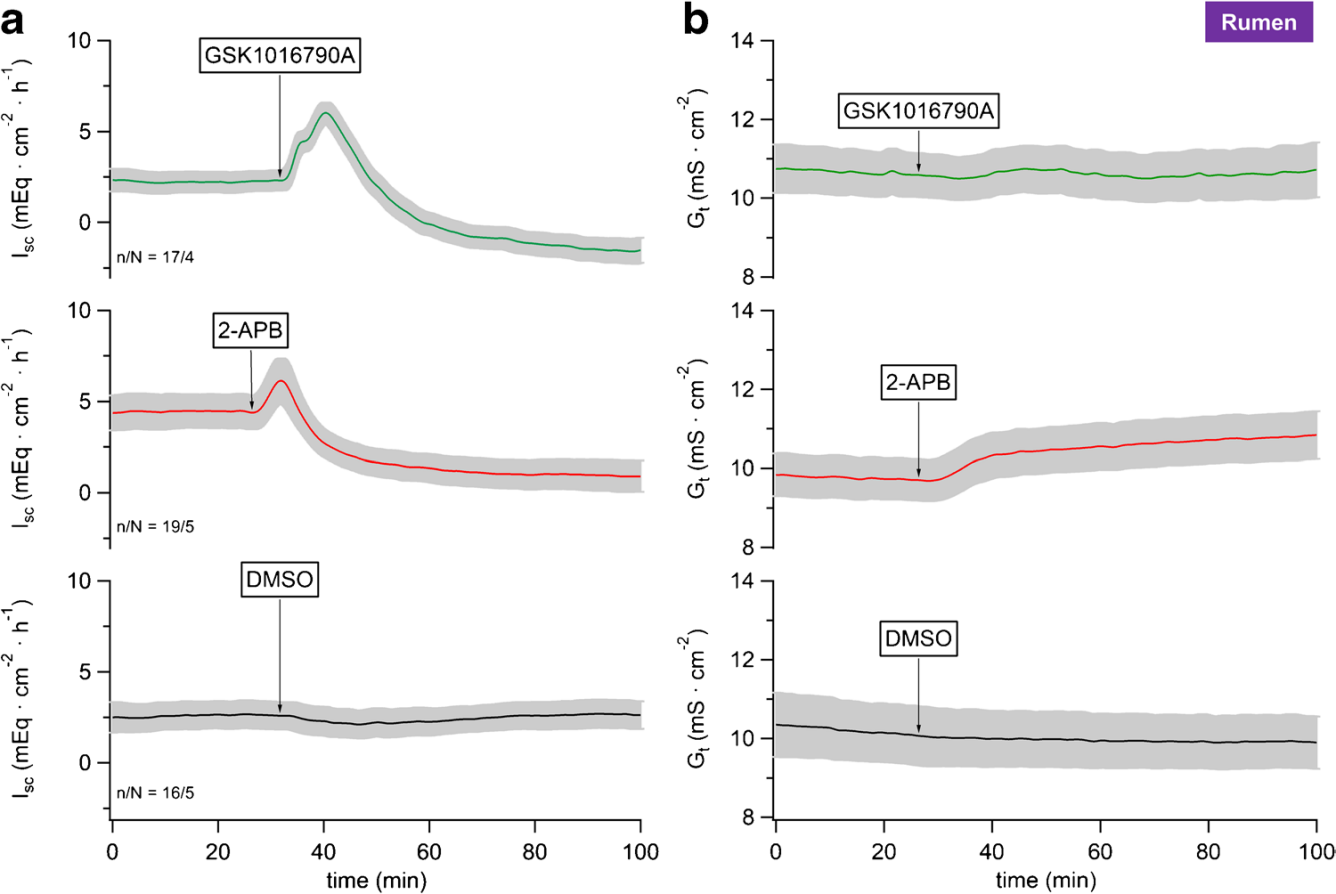
Ussing chamber measurements: native bovine ruminal epithelium. The mean *I*_sc_ (**a**) and the corresponding *G*_t_ (**b**) were plotted over time, both ± SEM (grey). After ~ 30 min, the agonists GSK1016790A (green, 2 µmol · L^−1^), 2-APB (red, 500 µmol · L^−1^), or an equivalent amount of solvent DMSO (black) were applied. The number of tissues (*n*) and the number of animals (*N*) are indicated in the figure. An rise in I_sc_ reflects an increase in cations transported from the mucosal to the serosal side

All ruminal tissues were superfused with physiological NaCl Ringer solutions (see methods) and equilibrated until the short-circuit current (*I*_sc_), and the conductance (*G*_t_) levels were stable. Initially, all tissues showed similar *I*_sc_ and *G*_t_ (Table 1). Subsequently, either GSK1016790A (0.2 or 2 µmol · L^−1^), 2-APB (500 µmol · L^−1^), or an equivalent amount of the solvent DMSO (1:1000, control) was added to the mucosal buffer. At 0.2 µmol · L^−1^, GSK1016790A showed no effect (*n*/*N* = 5/1, data not shown). At 2 µmol · L^−1^, GSK1016790A triggered a transient increase in *I*_sc_ (“peak”) followed by a decrease and stabilizing at a plateau, while *G*_t_ did not change significantly (Fig. 7 and Table 1). Similar changes in *I*_sc_ were visible after application of 2-APB although here, *G*_t_ increased continuously to a plateau level that was significantly higher than initially observed. The effects of 2-APB on *I*_sc_ and *G*_t_ are very similar to those observed in a previous study investigating other TRPV3 agonists on ruminal epithelia [78]. Control tissues showed no response (Fig. 7 and Table 1).

**Table 1:** Ussing chamber measurements: native bovine ruminal epithelium. Tissues were treated with either 2-APB (500 µmol · L^−1^), GSK1016790A (2 µmol · L^−1^), or an equivalent amount of solvent (control, 1:1000). Data are given as means ± SEM. The values in the “untreated” column reflect the short-circuit current (*I*_sc_) and the transcellular conductance (*G*_t_) measured 10 min prior to addition of the agonist. The maximum *I*_sc_ in the 15-min interval after treatment is given in the “peak” column. The “60-min” column gives *I*_sc_ and *G*_t_ measured 60 min after each addition. Superscripts within a row indicate significant differences (*p* ≤ 0.05) within one experimental group. The *p* values below reflect the results comparing all groups (ANOVA), followed by pairwise testing. The number of tissues are indicated by *n* and the number of cattle by *N*

*I*_sc_ (mEq · cm^−2^ · h^−1^)				
Treatment	Untreated	Peak	60 min	*n*/*N*
GSK1016790A	2.2 ± 0.6^a^	7.8 ± 1.7^b^	–1.4 ± 0.6^c^	17/4
2-APB	4.8 ± 0.9^a^	7.0 ± 1.2^b^	1.4 ± 0.9^c^	19/5
ctrl	2.6 ± 0.8^a^	2.8 ± 0.8^a^	2.6 ± 0.8^a^	16/5
	*p*_1_	*p*_2_	*p*_3_	
ANOVA	0.12	0.016	0.002	
GSK vs ctrl	0.6	0.011	≤ 0.001	
2-APB vs ctrl	0.2	0.015	0.3	
2-APB vs GSK	0.05	0.9	0.016	
*G*_t_ (mS · cm^−2^)				
Treatment	Untreated	(No peak)	60 min	*n*/*N*
GSK1016790A	10.6 ± 0.6^a^		10.7 ± 0.7^a^	17/4
2-APB	10.0 ± 0.7^a^		11.0 ± 0.7^b^	19/5
ctrl	10.2 ± 0.8^a^		9.9 ± 0.7^a^	16/5
	*p*_1_		*p*_3_	
ANOVA	0.7		0.4	
GSK vs ctrl	0.6		0.6	
2-APB vs ctrl	0.9		0.16	
2-APB vs GSK	0.4		0.5

Since no chemical gradient was present across the tissues, the increase in *I*_sc_ observed after application of these TRP agonists must reflect transcellular transport. Theoretically, the increase in *I*_sc_ might involve a tightening of the paracellular pathway. However, *G*_t_ either rose (2-APB) or remained the same (GSK1016790A), so that this explanation can be ruled out. Most likely, the agonists opened apical TRP channels with influx of Na^+^ (and small amounts of Ca^2+^), leading to the observed *I*_sc_ peak. The apical depolarization should stimulate efflux of K^+^, leading to the observed *I*_sc_ decrease, with current level finally stabilizing at a plateau with equal influx of Na^+^ and efflux of K^+^. In the case of 2-APB, the subsequent decrease in *I*_sc_ can additionally reflect an opening of the paracellular pathway. In the case of GSK1016790A, *G*_t_ remained the same so that this explanation is not an option.

## Studies of bTRPV4 and bTRPV3 HEK-293 cells in the whole-cell configuration of the patch-clamp technique

Previous studies from our laboratory suggest a role for bTRPV3 in the ruminal absorption of cations such as Na^+^, NH_4_^+^, and Ca^2+^ and in the secretion of K^+^ [56, 78, 79]. Since bTRPV4 may also contribute, we investigated the properties of this channel in comparison to bTRPV3 in overexpressing HEK-293 cells using the patch-clamp technique.

### *NaCl solution*

Functional expression of bTRPV4 by HEK-293 cells was confirmed via patch-clamp experiments with NaGlu in the pipette and in NaCl bath solution applying known TRPV4 modulators (Supplement, Part D, I) [107]. The concentration of Na^+^ was equal on both sides of the membrane with a Nernst equilibrium potential (*V*_eq_) of 0 mV, while Cl^−^ concentrations result in a calculated *V*_eq_ of − 51.05 mV.

In NaCl, the outward current of bTRPV4 cells was 24 ± 12 pA ∙ pF^−1^ at a pipette potential of + 100 mV, and the inward current was − 29 ± 14 pA ∙ pF^−1^ at − 120 mV, while the reversal potential (*V*_rev_) was 8 ± 4 mV (*n* = 12). Mean capacitance was 10.5 ± 2.1 pF and the mean series resistance 8.1 ± 1.5 MΩ. The positive *V*_rev_ argues for a contribution of Ca^2+^ to total permeability. The application of GSK1016790A (50 nmol · L^−1^) led to a significant rise in inward and outward currents. Outward current rose to 226 ± 56 pA ∙ pF^−1^ at + 100 mV (*p* ≤ 0.001), and inward current rose to − 220 ± 49 pA ∙ pF^−1^ (*p* ≤ 0.001) at − 120 mV, reflecting increases in the efflux and influx of Na^+^. The *V*_rev_ dropped slightly to 0.8 ± 2.3 mV (*p* = 0.042), close to the *V*_eq_ for Na^+^. Addition of the TRPV4 antagonist GSK2193874 (1 µmol · L^−1^) partially reversed the current effects. At + 100 mV, the current amplitude decreased significantly (177 ± 34 pA ∙ pF^−1^; *p* = 0.04), while at − 120 mV, the decrease was just numerical (− 176 ± 32 pA ∙ pF^−1^; *p* = 0.11). *V*_rev_ did not change (*p* = 0.3). Washout in NaCl was partial, with currents at 148 ± 30 pA ∙ pF^−1^ (at + 100 mV) and − 159 ± 34 pA ∙ pF^−1^ (at − 120 mV), while *V*_rev_ remained at 1 ± 4 mV (*p* = 0.6).

### *NH*_*4*_*Cl solution*

**Fig. 8:**
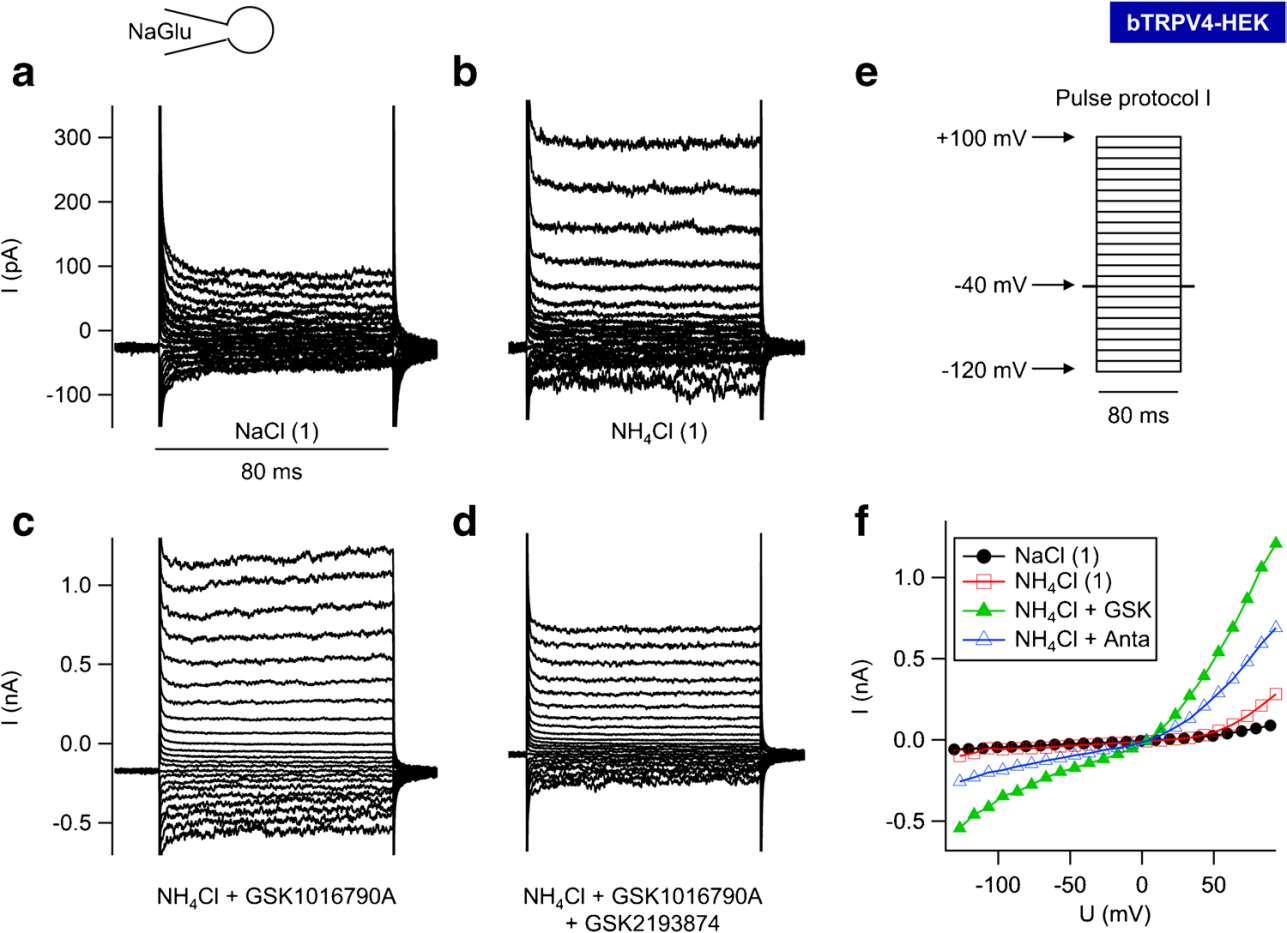
Whole-cell recording: bTRPV4 HEK-293 cell exposed to NH_4_Cl solution. This experiment was performed with a NaGlu pipette solution and stimulated by the pulse protocol I (**e**). **a** Currents in NaCl bath solution. **b** A switch to extracellular NH_4_Cl led to an enhanced current response at negative potentials (influx of NH_4_^+^) and positive potentials (efflux of Na^+^). **c** GSK1016790A (50 nmol · L^−1^) led to a significant increase in both inward and outward currents. Note the larger scaling. **d** Application of GSK2193874 (1 µmol · L^−1^) in continued presence of GSK1016790A partially reduced current level. **f** Current–voltage plot of the traces in **a** to **d**. Currents after stimulation with GSK1016790A (“GSK”, green-filled triangles) were partially reduced after additional application of GSK2193874 (“Anta”, blue blank triangles). Note the outward rectification with much higher currents at + 100 mV than at − 100 mV

**Fig. 9:**
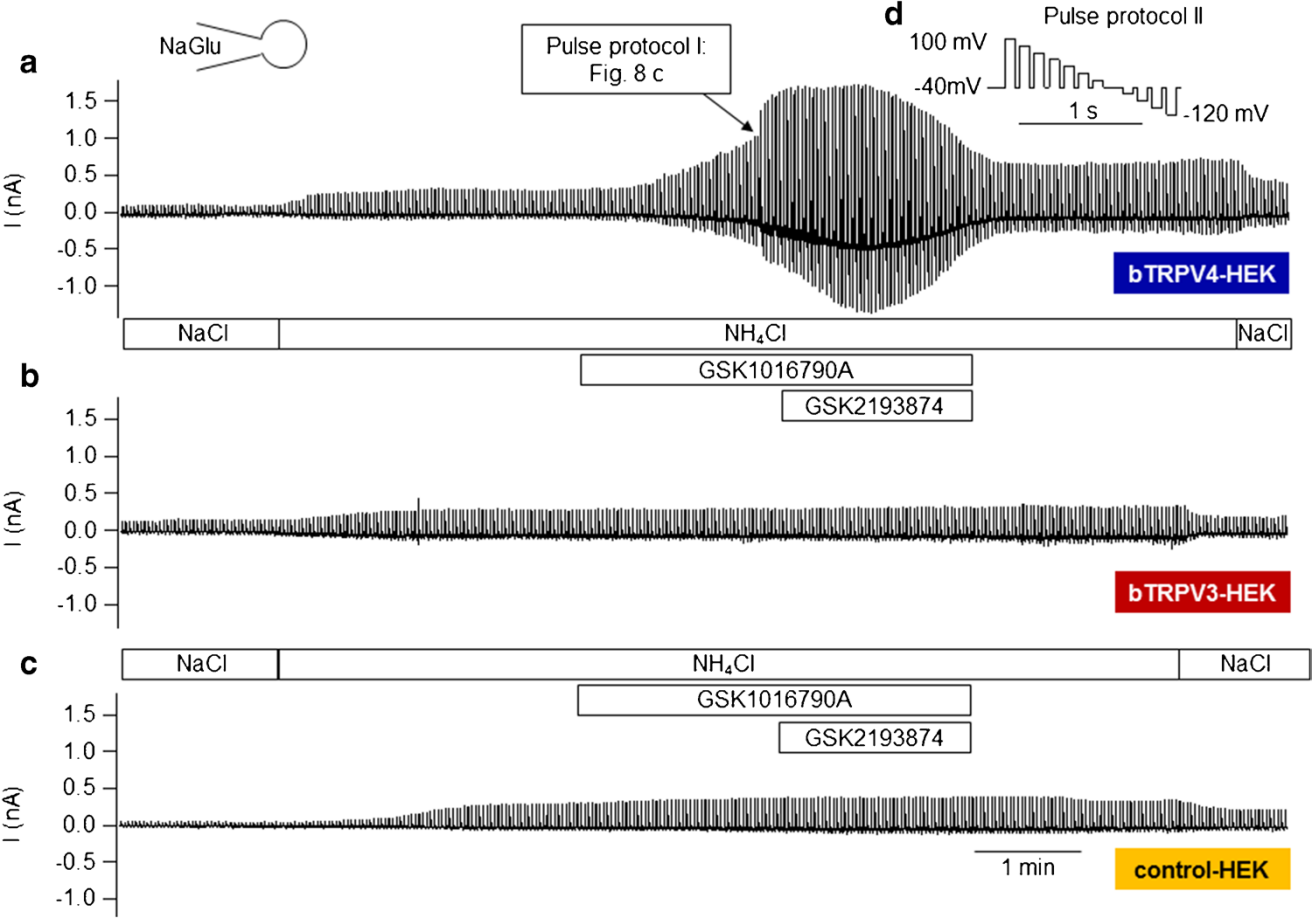
Whole-cell recordings: bTRPV3, bTRPV4, and control HEK-293 cells exposed to NH_4_Cl solution. Experiments were performed with a NaGlu pipette solution. Shown are the merged original recordings of bTRPV4 (**a**, same cell as in Fig. 8), bTRPV3 (**b**), and control HEK-293 cells (**c**) obtained by stimulating the cells with pulse protocol II (**d**). Intermittently, pulse protocol I (Fig. 8e) was applied, occasionally leading to visible gaps (arrow in **a**). Bath solutions are indicated by the bars. GSK1016790A (50 nmol · L^−1^) stimulates currents of bTRPV4 cells, with inhibitory effects of GSK2193874 (1 µmol · L^−1^). Cells expressing bTRPV3 or controls show a conductance to NH_4_^+^, but were not affected by GSK1016790A

To test for permeability of bTRPV4 to NH_4_^+^, HEK-293 cells expressing bTRPV4, bTRPV3, and controls were investigated in parallel (Table 2), again using a NaGlu pipette solution, and superfused with NaCl and then NH_4_Cl bath solutions (Supplement, Part D, II). Stimulation switched between the pulse protocols I (Fig. 8e) and II (Fig. 9d), with the latter merged to yield Figs. 9a, b, and c.

**Table 2:** Whole-cell recordings: bTRPV3, bTRPV4, and control HEK-293 cells in NH_4_Cl solution. bTRPV4 (V4), bTRPV3 (V3), and control (ctrl) HEK-293 cells were filled with a NaGlu pipette solution and superfused with solutions (Supplement, Part D, II) to which GSK1016790A (“GSK”, 50 nmol · L^−1^) and GSK2193874 (“Anta”, 1 µmol · L^−1^) were added (left column). The numbers in parentheses indicate consecutive applications of the same solution. Data are given as means ± SEM. The superscripts indicate significant differences (p ≤ 0.05) within each group or column. The *p* values obtained via ANOVA (all three groups) and via pairwise testing between the groups are given in the subsequent columns. The number of cells (*n*) in each group is given in the column headings. Positive currents reflect cations flowing out of the cell into the bath solution

NaGlu pipette/bath	bTRPV4 (*n* = 10)	Control (*n* = 8)	bTRPV3 (*n* = 7)	ANOVA (all)	*p*_1_ V4/ctrl	*p*_2_ V4/V3	*p*_3_ V3/ctrl
Current density at + 100 mV (pA ∙ pF^−1^)							
NaCl (1)	48 ± 19^a^	17 ± 5^a^	9 ± 3^a^	0.3	0.9	0.27	0.13
NH_4_Cl (1)	58 ± 20^b^	32 ± 7^b^	17 ± 3^b^	0.4	0.9	0.5	0.1
NH_4_Cl + GSK	286 ± 68^c^	33 ± 7^b^	20 ± 4^b^	0.002	0.009	0.004	0.1
NH_4_Cl + Anta	271 ± 63^c^	38 ± 8^c^	23 ± 5^b^	0.001	0.007	0.003	0.13
NH_4_Cl (2)	279 ± 65^c^	39 ± 9^c^	28 ± 7^c^	0.003	0.007	0.005	0.4
NaCl (2)	224 ± 49^d^	29 ± 8^b^	22 ± 7^b^	0.005	0.011	0.007	0.4
Current density at − 120 mV (pA ∙ pF^−1^)							
NaCl (1)	− 47 ± 20^a^	− 17 ± 6^a^	− 7.5 ± 2.6^a^	0.5	0.7	0.27	0.4
NH_4_Cl (1)	− 64 ± 24^a^	− 29 ± 9^b^	− 13 ± 4^b^	0.5	0.8	0.4	0.4
NH_4_Cl + GSK	− 332 ± 80^b^	− 32 ± 10^b^	− 16 ± 5^c^	0.004	0.009	0.007	0.24
NH_4_Cl + Anta	− 315 ± 75^b^	− 38 ± 11^b^	− 16 ± 5^c^	0.004	0.011	0.007	0.16
NH_4_Cl (2)	− 322 ± 77^b^	− 39 ± 12^b^	− 21 ± 6^d^	0.005	0.011	0.007	0.4
NaCl (2)	− 232 ± 55^c^	− 30 ± 9^b^	− 17 ± 8^b^	0.007	0.015	0.01	0.29
Reversal potential (mV)							
NaCl (1)	− 5 ± 9^a^	− 7 ± 5^a^	13 ± 10^a^	0.3	0.7	0.3	0.13
NH_4_Cl (1)	5 ± 7^a^	8 ± 4^b^	26 ± 6^a^	0.04	0.7	0.04	0.014
NH_4_Cl + GSK	3.0 ± 2.3^a^	11 ± 4^b^	25 ± 5^a^	0.005	0.12	0.005	0.08
NH_4_Cl + Anta	3.6 ± 2.2^a^	11 ± 4^b^	24 ± 5^a^	0.003	0.2	0.002	0.08
NH_4_Cl (2)	3.3 ± 2.4^a^	11 ± 3^b^	22 ± 4^a^	0.004	0.3	0.004	0.04
NaCl (2)	− 10 ± 4^b^	0 ± 3^a^	11 ± 6^b^	0.022	0.2	0.013	0.13
Relative permeability ratio p(NH_4_^+^)/p(Na^+^)							
NH_4_Cl (1)	1.6 ± 0.2^a^	2.0 ± 0.4^a^	2.0 ± 0.5^a^	0.7	0.4	0.6	0.9
NH_4_Cl + GSK	2.1 ± 0.7^a^	2.3 ± 0.6^a^	2.0 ± 0.5^a^	0.5	0.17	0.7	0.7

Currents of bTRPV4 cells were outwardly rectifying in NaCl and in NH_4_Cl solution (Fig. 8f), most likely reflecting block by extracellular Ca^2+^ and Mg^2+^ [71]. In all three groups, inward currents (at − 120 mV) increased in NH_4_Cl with a rise of *V*_rev_ (Fig. 8 and 9, Table 2), reflecting influx of NH_4_^+^. The increase in outward current (at + 100 mV) should reflect efflux of Na^+^ and/or influx of Cl^−^ induced by swelling, pH changes, or the classical stimulatory effect of a permeant ion on channel conductance [40].

Differences between the groups became significant after application of GSK1016790A (50 nmol · L^−1^) in NH_4_Cl bath. Both inward and outward currents of bTRPV4 cells rose significantly in contrast to those of control or bTRPV3 cells (Fig. 8c, f, Fig. 9). Some bTRPV4 cells responded strongly to the TRPV4 antagonist GSK2193874 (1 µmol · L^−1^) (Fig. 8d, f, 9a), but overall, effects were limited and did not test for significance (Table 2). Possibly, ongoing activation of current by NH_4_Cl obscured the GSK2193874 effect. Partial washout could be observed.

After the first solution change, *V*_rev_ of bTRPV3 cells was significantly higher than that of bTRPV4 cells or controls, the latter not having been previously observed [79]. A possible reason might be the absence of Mg^2+^ in the pipette solution of our current study, leading to a higher influx of Ca^2+^.

In all groups, the permeability of NH_4_^+^ was greater than that of Na^+^ (Table 2; for calculation, see Supplement, Part F). This suggests involvement of poorly selective channels with permeability determined by the energy required for the removal of the hydration shell of the permeating cations [73]. In the case of bTRPV3, data were in good agreement with the ratio that can be calculated from the single-channel conductance for NH_4_^+^ (~ 240 pS) and Na^+^ (~ 128 pS) from a previous inside-out patch-clamp study [56, 79].

In conjunction, bTRPV4 channels conduct Na^+^, but have an even higher permeability to NH_4_^+^. Furthermore, the TRPV4 agonist GSK1016790A strongly stimulated bTRPV4, but had no effects on bTRPV3 or control cells, which is not trivial.

### *K-Gluconate pipette solution*

**Fig. 10:**
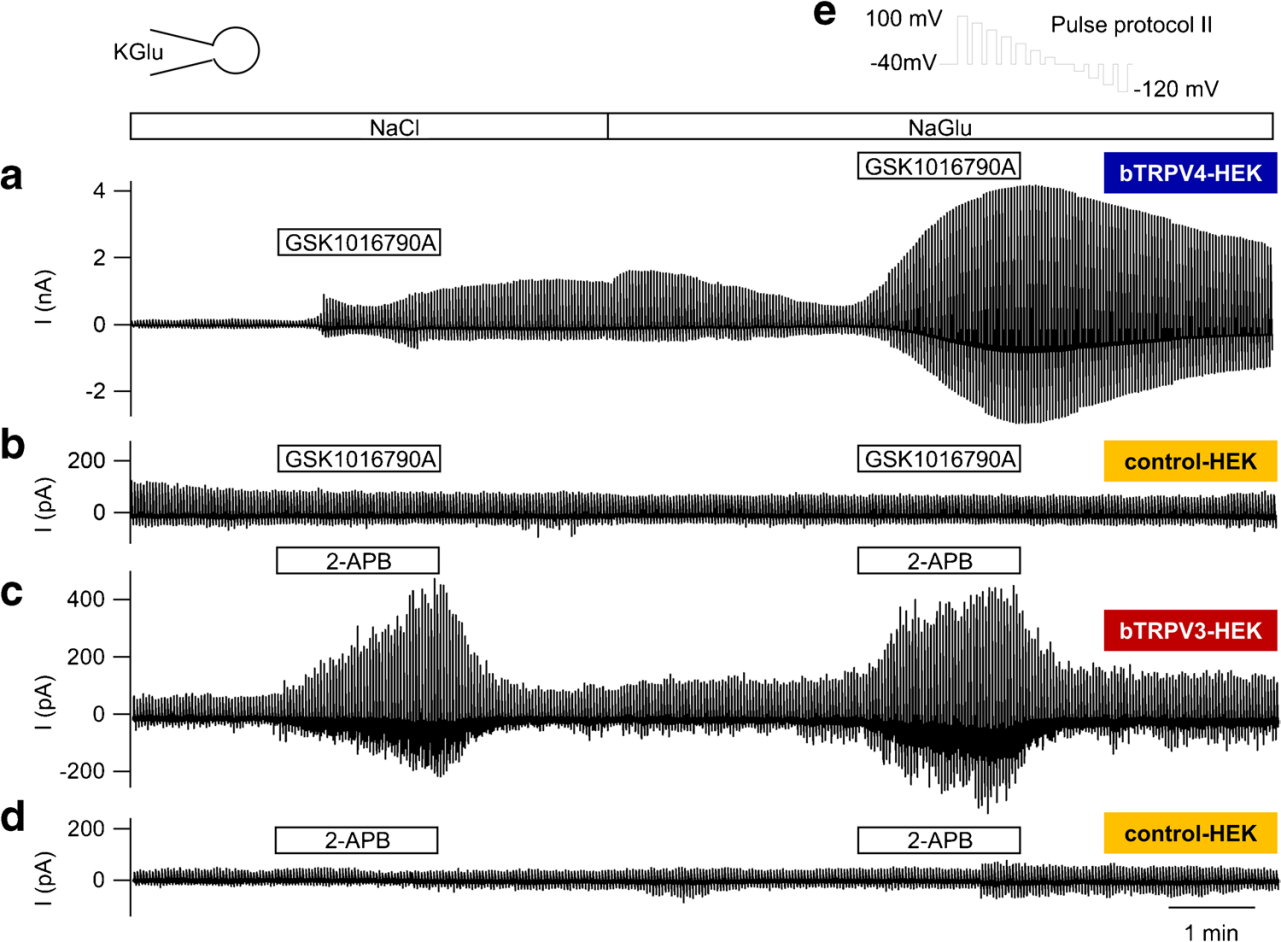
Whole-cell recordings: bTRPV3, bTRPV4, and control HEK-293 cells filled with K-Gluconate solution. Experiments were performed with a KGlu pipette solution and initial in a NaCl bath. Shown are the merged original recordings of bTRPV4 (**a**), control (**b**), bTRPV3 (**c**), and control HEK-293 cells (**d**) obtained by stimulating the cells with protocol II (**e**). To rule out a possible participation of Cl^−^ in the response, cells were superfused with NaGlu solution in the latter half of the experiment, as indicated by the bar at the top. bTRPV4 HEK-293 cells showed a pronounced response after exposition to GSK1016790A (**a**) in contrast to unresponsive controls (**b**), requiring a different scale. Using 2-APB, bTRPV3 currents were significantly stimulated (**c**) in contrast to controls (**d**)

The Ussing chamber experiments suggest that GSK1016790A (2 µmol · L^−1^) and 2-APB (500 µmol · L^−1^) stimulated not only influx of Na^+^ but also efflux of K^+^, resulting in a biphasic *I*_sc_ response. To test this hypothesis, whole-cell experiments with a KGlu pipette solution were performed with HEK-293 cells that overexpressed bTRPV3 or bTRPV4 (Fig. 10, Table 3). In addition to KGlu, the pipette solution contained Ca^2+^, Mg^2+^, and ATP (Supplement, Part D, III).

**Table 3:** Whole-cell recordings: bTRPV3, bTRPV4, and control HEK-293 cells filled with K-Gluconate pipette solution Cells were filled with a KGlu pipette solution and superfused with various solutions (Supplement, Part D, III) to which GSK1016790A (50 nmol · L) and 2-APB (300 µmol · L) were added as indicated in the left column. All else is essentially presented as in Table 2

Pipette KGlu	bTRPV4 (*n* = 10)	control (*n* = 8)	p_1_ V4/ctrl	p_2_ V3/V4	bTRPV3 (*n* =10)	control V3/ctrl	p_3_ V3/ctrl	ANOVA (all)
Bath	Current density at +100 mV (pA ∙ pF^-1^)							
NaCl (1)	33 ± 9^a^	10 ± 4^a^	0.007	0.006	10.3 ± 2.4^a^	11.5 ± 2.9^a^	0.8	0.008
NaCl + agonist	GSK1016790A				2-APB			
	178 ± 45^b^	9 ± 3^a^	≤ 0.001	0.004	52 ± 20^b^	11 ± 4^a^	0.009	≤0.001
NaCl (2)	168 ± 33^b^	9.1 ± 2.7^a^	≤ 0.001	≤0.001	17 ± 7^a^	13 ± 5^a^	0.28	≤0.001
NaGlu (1)	105 ± 11^c^	10 ± 3^a^	≤ 0.001	≤ 0.001	21 ± 7^a^	13 ± 6^a^	0.13	≤0.001
NaGlu + agonist	GSK1016790A				2-APB			
	555 ± 109^d^	11 ± 4^a^	≤ 0.001	≤ 0.001	87 ± 29^c^	14 ± 6^a^	0.004	≤0.001
NaGlu (2)	473 ± 63^d^	11 ± 4^a^	≤ 0.001	≤ 0.001	57 ± 29^b^	16 ± 6^a^	0.08	≤0.001
NaCl (3)	193 ± 28^e^	9.8 ± 2.7^a^	≤ 0.001	≤ 0.001	38 ± 17^a^	19 ± 8^a^	0.22	≤0.001
Current density at -120 mV (pA ∙ pF^-1^)								
NaCl (1)	-33 ± 9^a^	-4.6 ± 1.5^a^	0.001	0.003	-6.5 ± 1.9^a^	-5.4 ± 2.3^a^	0.4	0.001
NaCl + agonist	GSK1016790A				2-APB			
	-129 ± 37^b^	-5.1 ± 2.0^a^	≤ 0.001	≤ 0.001	-14 ± 5^a^	-6.9 ± 2.8^a^	0.11	≤0.001
NaCl (2)	-98 ± 23^c^	-4.5 ± 1.7^a^	≤ 0.001	≤ 0.001	-12 ± 5^a^	-8 ± 4^a^	0.28	≤ 0.001
NaGlu (1)	-73 ± 11^d^	-5.1 ± 2.1^a^	≤ 0.001	≤ 0.001	-14 ± 6^a^	-7 ± 4^a^	0.05	≤ 0.001
NaGlu + agonist	GSK1016790A				2-APB			
	− 387 ± 88^e^	− 5.3 ± 1.9^a^	≤ 0.001	≤ 0.001	− 55 ± 26^b^	− 8 ± 4^a^	0.013	≤ 0.001
NaGlu (2)	− 280 ± 41^f^	− 5.2 ± 1.7^a^	≤ 0.001	≤ 0.001	− 21 ± 8^a^	− 8 ± 4^a^	0.012	≤ 0.001
NaGlu (3)	− 211 ± 81^g^	− 5.3 ± 1.7^a^	≤ 0.001	0.002	− 30 ± 15^a^	− 8 ± 4^a^	0.014	≤ 0.001
Reversal potential (mV)								
NaCl (1)	− 5.2 ± 2.4^a^	− 25 ± 4^a^	≤ 0.001	0.05	−19 ± 6^a^	0.30 ± 6^a^	0.25	0.001
NaCl + agonist	GSK1016790A				2-APB			
	−3.4 ± 1.6^a^	−27 ± 5^a^	≤ 0.001	0.22	−7.7 ± 2.8^b^	−26 ± 7^a^	0.004	≤ 0.001
NaCI (2)	−2.3 ± 1.1^b^	−25 ± 5^a^	≤ 0.001	0.026	−15 ± 4^a^	−25 ± 5^a^	0.13	≤ 0.001
NaGlu (1)	-2.8 ± 1.5^b^	-23 ± 5^a^	0.001	0.06	-10 ± 4^b^	-23 ± 3^a^	0.014	≤ 0.001
NaGlu + agonist	GSK1016790A				2-APB			
	-6.2 ± 1.5^a^	-21 ± 5^b^	0.006	1.0	-6 ± 2^b^	-20 ± 4^a^	0.003	≤ 0.001
NaGlu (2)	-6.1 ± 1.4^a^	-20 ± 5^b^	0.006	0.5	-9 ± 4^b^	-23 ± 4^a^	0.013	0.001
NaCl (3)	-7.1 ± 1.8^a^	-24 ± 5^a^	≤ 0.001	0.4	-10 ± 4^a^	-29 ± 5^a^	0.013	≤ 0.001
Relative permeability ratio p(NH_4_^+^)/p(Na^+^)								
NaGlu (1)	1.23 ± 0.08^a^	3.1 ± 0.7^a^	0.001	0.06	1.72 ± 0.21^a^	2.9 ± 0.4^a^	0.014	≤ 0.001
NaGlu + agonist	GSK1016790A				2-APB			
	1.4 ± 0.1^b^	3.0 ± 0.7^b^	0.006	0.8	1.36 ± 0.11^a^	2.8 ± 0.6^a^	0.002	≤ 0.001
NaGlu (2)	1.40 ± 0.09^b^	2.9 ± 0.7^b^	0.006	0.7	1.58 ± 0.22^a^	2.9 ± 0.4^a^	0.007	≤ 0.001

Currents in bTRPV4 cells were significantly higher than in control or bTRPV3 cells even before application of an agonist (Table 3), possibly due to inclusion of ATP in the pipette solution [74]. GSK1016790A (50 nmol · L^−1^) significantly enhanced bTRPV4 currents at both positive and negative potentials with subsequent washout, while 2-APB (300 µmol · L^−1^) stimulated bTRPV3 currents. No effects were seen in the two control groups, which did not differ from each other (*p* > 0.3). In a previous study, agonist induced responses were decreased by NaGlu [74]. This could not be confirmed, most likely because TRP channels typically respond more strongly to the second application of an agonist than to its primary application [57, 60, 74]. In our experiments, the effects in NaGlu strongly suggest that both agonists stimulate efflux of K^+^ rather than influx of Cl^−^. Furthermore, replacement of Cl^−^ by the poorly permeable gluconate^−^ made it possible to estimate the relative permeability ratio for K^+^ relative to Na^+^ (Table 3). As expected of non-selective cation channels, the overexpressing cells were significantly less able to discriminate between Na^+^ and K^+^ than the controls.

### *Butyrate*^*−*^*solutions*

Microbial fermentation of plant material within the rumen releases large quantities of short-chain fatty acids (SCFA), which stimulate the transport of Na^+^ across native ruminal epithelia as shown in Ussing chamber experiments [2, 103]. This involves stimulation of an electroneutral mechanism (Na^+^/H^+^ exchange), but also stimulation of an electrogenic pathway [55], with the increase in *I*_sc_ reduced by concomitant activation of an H^+^-ATPase [52]. Furthermore, SCFA stimulate transport of ammonia [12, 13], and in cultured ruminal epithelial cells, we observed stimulation of cation currents by SCFA [33]. Both bTRPV3 and bTRPV4 are potential candidates for these effects. We investigated the response of HEK-293 cells overexpressing bTRPV3, bTRPV4, and controls to application of solutions containing the SCFA sodium butyrate (NaBu) (30 mmol ∙ L^−1^) [82] at a pH of 7.4 and at the pH of 6.4 that is physiologically found in rumen (Supplement, Part D, IV). Note that when pH drops from 7.4 to 6.4, the amount of protonated butyrate^−^ (butyric acid) increases tenfold from 0.25 to 2.45%.

**Fig. 11:**
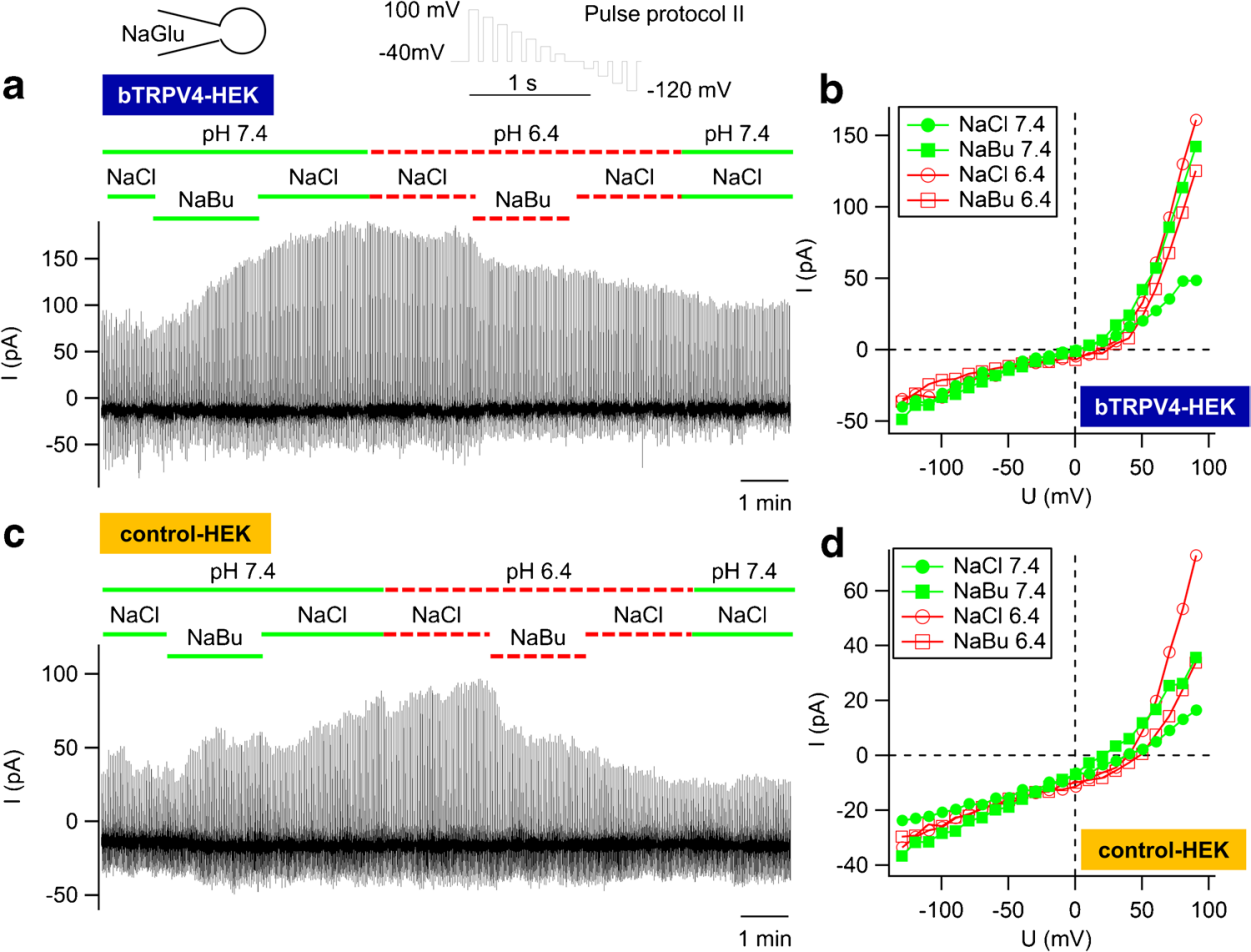
Whole-cell recordings: bTRPV4 and control HEK-293 cells in butyrate^−^ solution. Experiments were performed with a NaGlu pipette solution. A HEK-293 cell overexpressing bTRPV4 (**a**) and a control cell (**c**) were exposed to pulse protocol II, with the corresponding current–voltage plots in **b** and **d**. Cells were filled with NaGlu pipette solution and superfused with solutions as indicated at the top. After incubation in NaCl, cells were exposed to NaBu (30 mmol · L^−1^) at pH 7.4 (green) and pH 6.4 (red). The responses of individual cells were quite variable, and the rise in currents at positive potentials seen in response to NaBu 7.4 in **a** or **c** did not test for significance, in contrast to the subsequent current decrease in response to NaBu 6.4 in bTRPV4 group (see Table 4)

**Fig. 12:**
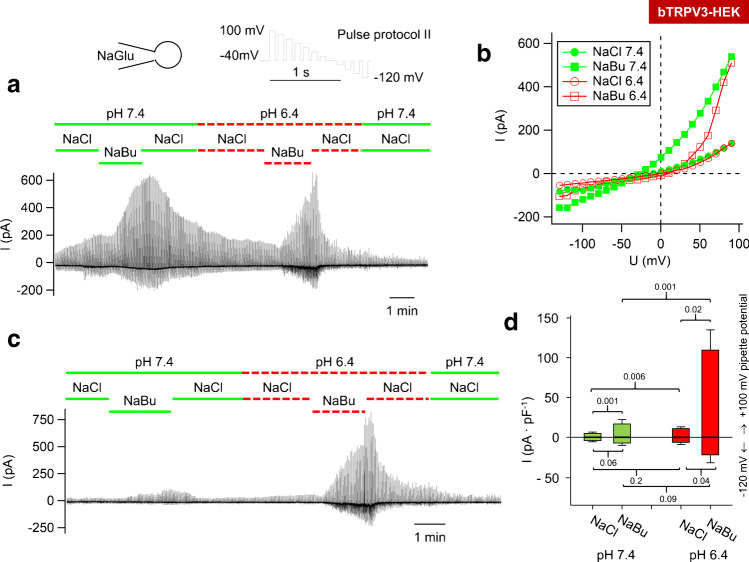
Whole-cell recording: bTRPV3 HEK-293 cells in butyrate^−^ solution. Experiments were performed with a NaGlu pipette solution. Data obtained by applying protocol II were merged. **a** In this cell, a large response to NaBu (30 mmol · L^−1^) could be seen, the magnitude of which did not depend on the pH. **b** The corresponding current–voltage (IV) plot of the cell in **a**. **c** This cell is more typical and showed a much larger response to NaBu at pH 6.4 than at pH 7.4. (For IV plot and current kinetics, see Fig. 13). **d** Comparison of the mean (± SEM) current responses to NaBu at + 100 and − 120 mV pipette potential. To rule out effects of a repeated exposure, the graph only includes data from the first exposure to NaBu. A total of *n* = 17 cells were exposed to NaCl and NaBu at pH 7.4 (green), while *n* = 7 cells were exposed to NaCl and NaBu at pH 6.4 (red). (For statistics of all data, see Table 4, and for solutions, Supplement, Part D, IV)

In control and bTRPV4 cells, the effects of NaBu tended to be small and variable (Table 4). After application of NaBu 7.4, a small increase in currents at positive potentials was seen in some bTRPV4 and control cells that persisted after washout with NaCl 7.4 and NaCl 6.4 (Fig. 11). Somewhat surprisingly, the subsequent addition of NaBu 6.4 induced a reduction of current in these cells (Fig. 11). In bTRPV4 cells, the decrease in inward current tested for significance (Table 4). Conversely, in bTRPV3 cells, a stimulatory effect of butyrate^−^ on inward and outward currents could be observed that was highly significant at the more acidic pH, with partial washout (Fig. 12). *V*_rev_ did not change significantly, although for unclear reasons, *V*_rev_ of bTRPV4 cells was higher than *V*_rev_ of controls or bTRPV3 cells, reaching significant *p* values in some bath solutions.

**Table 4:** Whole-cell recording: bTRPV3, bTRPV4, and control HEK-293 cells in butyrate^−^ solution. bTRPV3 (V3), bTRPV4 (V4), and control (ctrl) HEK-293 cells were filled with a NaGlu pipette solution and superfused with solutions as indicated in left column (Supplement, Part D, IV) in order to investigate the effect of NaBu (30 mmol ∙ L^−1^) at pH 7.4 and pH 6.4. The numbers in parentheses indicate consecutive applications of the same solution. The data represent means ± SEM. The superscripts indicate significant differences (*p* ≤ 0.05) within each group or column. The *p* values obtained via ANOVA (all three groups) and via pairwise testing between the groups are given in the last four columns. The number of cells (*n*) is given in the rows above each subset of data

NaGlu pipette/bath, pH	bTRPV4	control	bTRPV3	p_1_ V4/ctrl	p_2_ V4/V3	p_3_ V3/ctrl	ANOVA (all)
Current density at +100 mV (pA ∙ pF^-1^)							
	n = 12	n = 22	n = 17				
NaCl 7.4 (1)	5.8 ± 1.7^a^	7.2 ± 1.8^a^	5.3 ± 1.2^a^	1.0	0.7	0.6	0.4
NaBu 7.4	6.1 ± 1.3^a^	21 ± 11^a^	17 ± 5^b^	0.5	0.4	0.9	0.4
NaCl 7.4 (2)	7.8 ± 1.3^a^	17 ± 9^a^	11.1 ± 2.7^b^	0.6	0.9	0.7	0.5
	n = 11	n = 21	n = 22				
NaCl 6.4 (1)	8.7 ± 2.6^a^	17 ± 7^a^	9.1 ± 1.2^b^	0.19	0.5	0.6	0.26
NaBu 6.4	5.9 ± 2.2^a^	9.9 ± 2.1^a^	61 ± 13^c^	0.18	≤ 0.001	≤ 0.001	0.026
NaCl 6.4 (2)	4.8 ± 1.7^b^	9.5 ± 2.6^a^	51 ± 14^b^	0.5	0.002	0.007	0.20
Current density at -120 mV (pA ∙ pF^-1^)							
	n = 12	n = 22	n = 17				
NaCl 7.4 (1)	-6.1 ± 1.6^a^	-6.5 ± 1.5^a^	-4.7 ± 1.1^a^	0.8	0.26	0.23	0.9
NaBu 7.4	-5.5 ± 1.4^b^	-9.3 ± 2.7^a^	-7.9 ± 2.3^a^	0.25	1.0	0.29	0.6
NaCl 7.4 (2)	-5.7 ± 1.3^b^	-7.8 ± 1.9^a^	-5.6 ± 1.3^a^	0.4	0.7	0.28	0.9
	n = 11	n = 21	n = 22				
NaCl 6.4 (1)	-4.2 ± 1.3^c^	-7.6 ± 2.1^a^	-5.3 ± 0.9^a^	0.13	0.23	0.5	0.5
NaBu 6.4)	-4.1 ± 1.3^d^	-5.4 ± 1.0^a^	-21 ± 10^b^	0.17	0.014	0.1	≤ 0.001
NaCl 6.4 (2)	-4.1 ± 1.4^d^	-7.0 ± 2.0^a^	-18 ± 7^a^	0.18	0.09	0.6	0.002
Reversal potential (mV)							
	n = 12	n = 22	n = 17				
NaCl 7.4 (1)	19 ± 7^a^	6 ± 6^a^	-5 ± 9^ab^	0.19	0.08	0.5	0.19
NaBu 7.4	22 ± 5^a^	3 ± 5^a^	-6 ± 9^b^	0.012	0.016	0.17	0.012
NaCl 7.4 (2)	22 ± 5^a^	7 ± 5^a^	-2 ± 9^ab^	0.05	0.05	0.4	0.28
	n = 11	n = 21	n = 22				
NaCl 6.4 (1)	17 ± 7^a^	7 ± 7^a^	-2 ± 7^a^	0.4	0.05	0.5	0.18
NaBu 6.4	20 ± 8^a^	12 ± 10^a^	0 ± 5^a^	0.7	0.006	0.5	0.10
NaCl 6.4 (2)	16 ± 9^a^	2 ± 9^a^	3 ± 6^a^	0.16	0.14	0.8	0.26

**Fig. 13:**
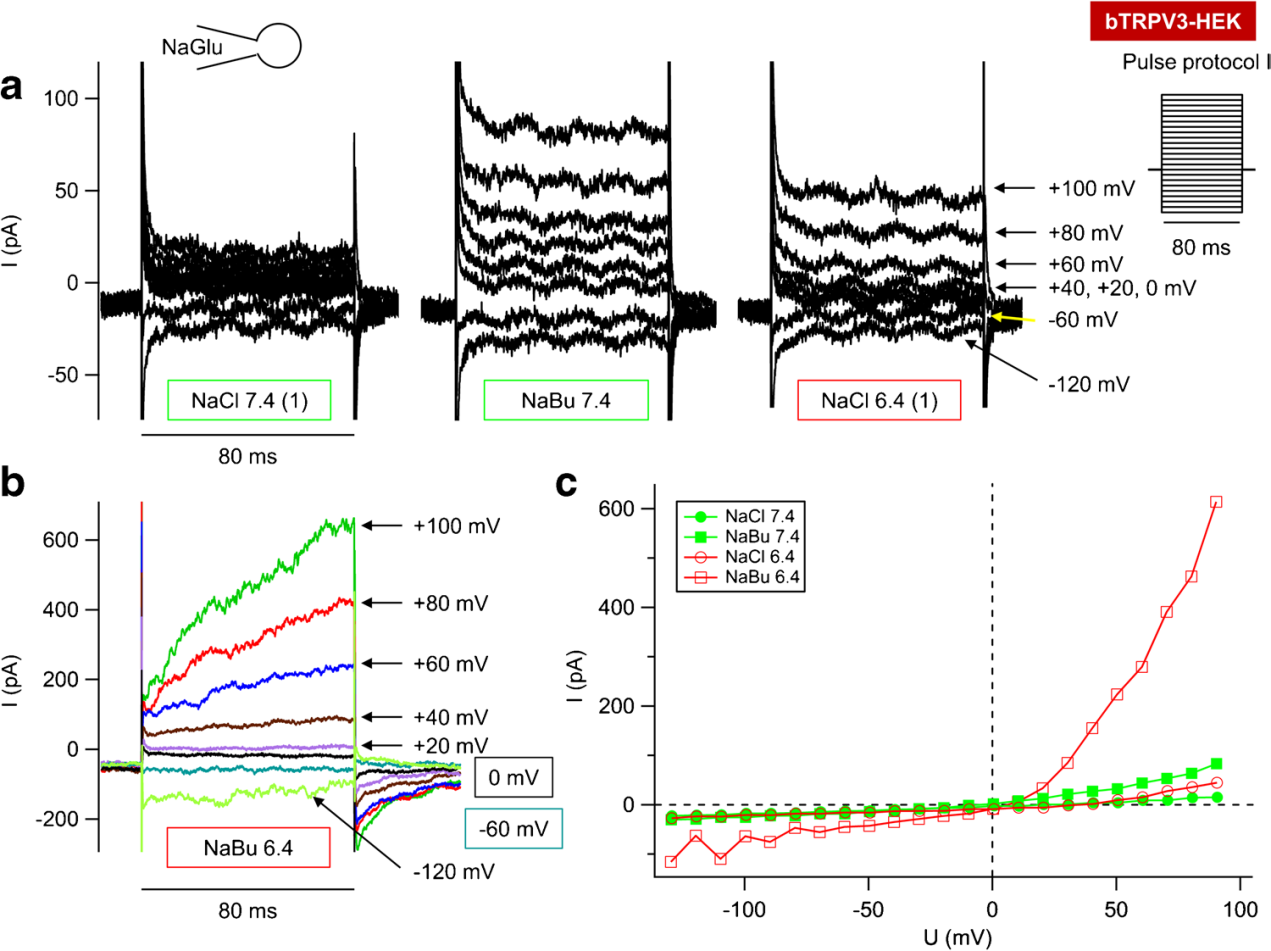
Whole-cell recording: bTRPV3 HEK-293 cell in butyrate^−^ solution. The kinetics of currents obtained with pulse protocol I (**a**, **b**) and corresponding current–voltage plot (**c**) are displayed for the bTRPV3 HEK-293 cell (same cell as in Fig. 12c). For clarity, only traces for the indicated voltage steps are shown (arrows). **a** The current responses in NaCl 7.4, NaBu 7.4 and NaCl 6.4 were small (< 100 pA), and currents did not show time-dependent activation. **b** A change to NaBu at pH 6.4 induced higher currents (higher scaling). Note the time-dependent current increases after each depolarization and the pronounced tail currents after a return to − 40 mV

In total, 15 bTRPV3 cells survived exposure not only to NaBu 7.4 but also to NaBu 6.4 (Fig. 12). In some cells, pH had no effect on the NaBu response (Fig. 12a), while other cells responded more strongly to the second application at pH 6.4 (Fig. 12c). The butyrate^−^-activated currents showed strong outward rectification (Fig. 12b, 13c), time-dependent activation, and pronounced tail currents (Fig. 13b), most likely reflecting the effects of a voltage-dependent block by divalent cations. Since the greater effects of NaBu 6.4 might be due to the prior stimulation of bTRPV3 as observed above for 2-APB, further 7 cells were directly exposed to NaCl 6.4 and NaBu 6.4 without pretreatment. Compared to the 17 cells initially exposed to NaBu 7.4, effects of NaBu were greater at pH 6.4 than at pH 7.4 (Fig. 12d).

A possible hypothesis is that influx of butyric acid caused volume changes. However, the capacitance of cells expressing bTRPV3 dropped slightly during the application of butyrate^−^, with values at 14.8^a^ ± 1.7 pF (NaCl 7.4), 14.6^a^ ± 1.7 pF (NaBu 7.4), 14.5^b^ ± 1.7 pF (NaCl 7.4) for *n* = 17, 14.9^c^ ± 1.5 pF (NaCl 6.4), 13.2^c^ ± 1.0 pF (NaBu 6.4), and 12.7^c^ ± 1.0 pF (NaCl 6.4) for *n* = 22, where different superscripts designate significant differences (ANOVA on ranks). Values for control cells (in pF and same order as above) are 12.4^a^ ± 1.7, 12.4^a^ ± 1.6, 12.2^a^ ± 1.6 (for *n* = 22), 12.9^b^ ± 1.7, 13.1^b^ ± 1.6, and 12.9^c^ ± 1.6 (for *n* = 21). Most likely, back flux into the pipette prevented any cell swelling, and the changes are spurious.

The capacitances of bTRPV4 cells were marginally lower (*p* ≤ 0.05, ANOVA on ranks) than those of bTRPV3 cells or controls (*p* > 0.2, controls versus bTRPV3), with a slight tendency to rise during the experiment (9.1^a^ ± 1.0, 9.1^a^ ± 1.0, 8.9^a^ ± 0.9 for *n* = 12, 9.4^b^ ± 0.7, 9.4^c^ ± 0.7, 9.2^b^ ± 0.7 for *n* = 11). Most likely, this difference is spurious although it is also tempting to speculate that the outstanding ability of TRPV4 to sense changes in cell volume played a role [99]. Volume regulation might also have influenced the very variable outcomes of experiments on individual cells.

## Effect of butyrate^−^ on intracellular Ca^2+^ ([Ca^2+^]_i_)

SCFA stimulate the ruminal absorption of Ca^2+^ in vitro and in vivo [82, 83, 104, 110]. To investigate possible uptake mechanisms, HEK-293 cells transfected with p5TO-*bTRPV4*, p5TO-*bTRPV3*, or the empty p5TO vector (controls) were examined using an intracellular calcium fluorescence imaging technique allowing measurements on single cells. The solutions had a pH of 6.4 and were otherwise identical to those above (Supplement, Part D, IV).

**Fig. 14:**
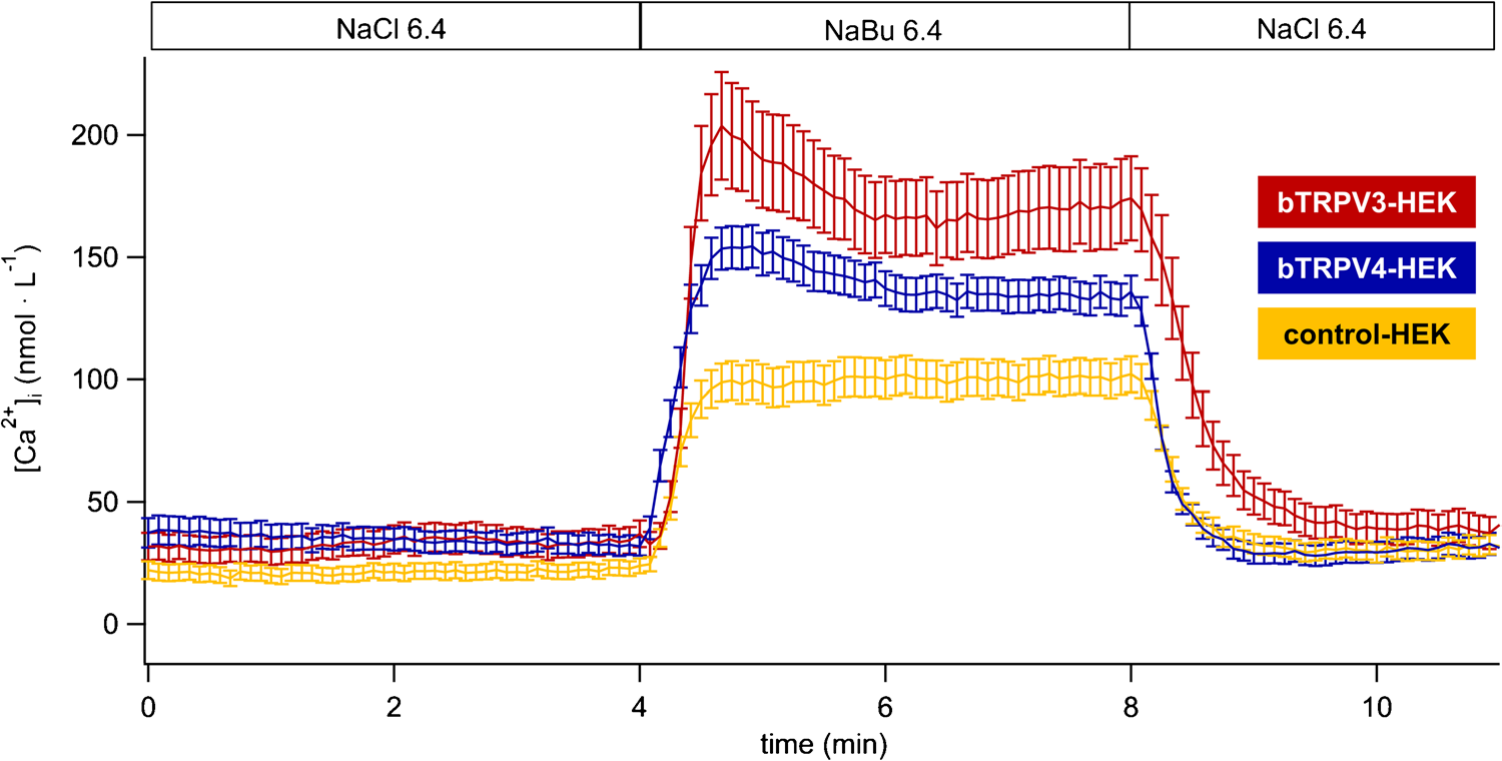
Intracellular calcium fluorescence imaging: bTRPV3, bTRPV4, and control HEK-293 cells in butyrate^−^ solution. The figure shows means ± SEM of [Ca^2+^]_i_ in HEK-293 cells overexpressing bTRPV3 (*n*/*N* = 62/5), bTRPV4 (*n*/*N* = 145/11), and control HEK-293 cells (*n*/*N* = 143/9, *n*: number of cells, *N*: number of coverslips) illustrating the significantly higher effect of NaBu 6.4 on bTRPV3 and bTRPV4 than on control cells

In initial NaCl 6.4, all three groups showed similar [Ca^2+^]_i_, suggesting efficient mechanisms for regulating Ca^2+^ influx and efflux (Fig. 14, Table 5). Significant [Ca^2+^]_i_ increases were observed in all three groups after exposure to NaBu 6.4. However, effects were significantly higher in bTRPV3 and bTRPV4 cells than in controls, with no significant difference between bTRPV3 and bTRPV4 (Table 5). Interestingly, in both bTRPV3 and bTRPV4 HEK-293 cells, [Ca^2+^]_i_ rose within 1 min after application of NaBu 6.4 to a “peak” and subsequently dropped slightly to a lower plateau, which may reflect partial block of the channel after binding of Ca^2+^ to a calmodulin binding domain (Fig. 14) [74, 113]. In contrast, [Ca^2+^]_i_ of controls remained constant after the initial increase (Fig. 14).

**Table 5:** Intracellular calcium fluorescence imaging: bTRPV3, bTRPV4, and control HEK-293 cells in butyrate^−^ solution. bTRPV3 (V3), bTRPV4 (V4), and control (ctrl) HEK-293 cells were loaded with fura-2. Cells were superfused with solutions as indicated in left column (Supplement, Part D, IV) to investigate the effect of NaBu (30 mmol ∙ L^−1^) at pH 6.4. Data are given as means ± SEM and were obtained 3.5 min in each solution, with the exception of NaBu 6.4 (peak), which reflects the maximal change measured within the first minute after the switch to NaBu 6.4. The superscripts indicate significant differences (*p* ≤ 0.05) within each group or column. The *p* values obtained via ANOVA (all three groups) and via pairwise testing between the groups are given in the last four columns. The number of individual cells and coverslips is given in brackets (*n*/*N*) in the column headings

Intracellular calcium concentration [Ca^2+^]_i_ (nmol ∙ L^-1^)							
Bath, pH	bTRPV3 (62/5)	bTRPV4 (145/11)	control (143/9)	ANOVA (all)	*p*_1_ V4/ctrl	*p*_2_ V4/V3	*p*_3_ V3/ctrl
NaCl 6.4 (1)	33 ± 6^a^	33 ± 5^a^	21 ± 3^a^	0.2	0.1	0.6	0.1
NaBu 6.4 (peak)	190 ± 20^b^	151 ± 9^b^	98 ± 8^b^	≤0.001	≤ 0.001	0.5	≤ 0.001
NaBu 6.4 (plateau)	170 ± 18^c^	135 ± 7^c^	101 ± 8^c^	≤0.001	≤ 0.001	0.5	≤ 0.001
NaCl 6.4 (2)	38 ± 7^a^	31 ± 5^a^	31 ± 4^d^	0.7	0.4	0.9	1.0

## Discussion

In previous studies of ruminal transport, it has emerged that many of the transport proteins typically expressed by gastrointestinal epithelia such as ENaC, CFTR, TRPV5, or TRPV6 are not involved [55]. Instead, the tissue expresses a non-selective, divalent-sensitive cation conductance that physiologically serves as a route for the uptake of Na^+^, Ca^2+^, and NH_4_^+^. A similar, amiloride-insensitive cation conductance has previously been observed in the omasum [84], in rabbit and rat cecum [75, 86], in the proximal colon of rats [29], and in colon of *Xenopus laevis* frogs [51]. There is also evidence for a similar pathway in the colon and cecum of pigs [61].

**Fig. 15:**
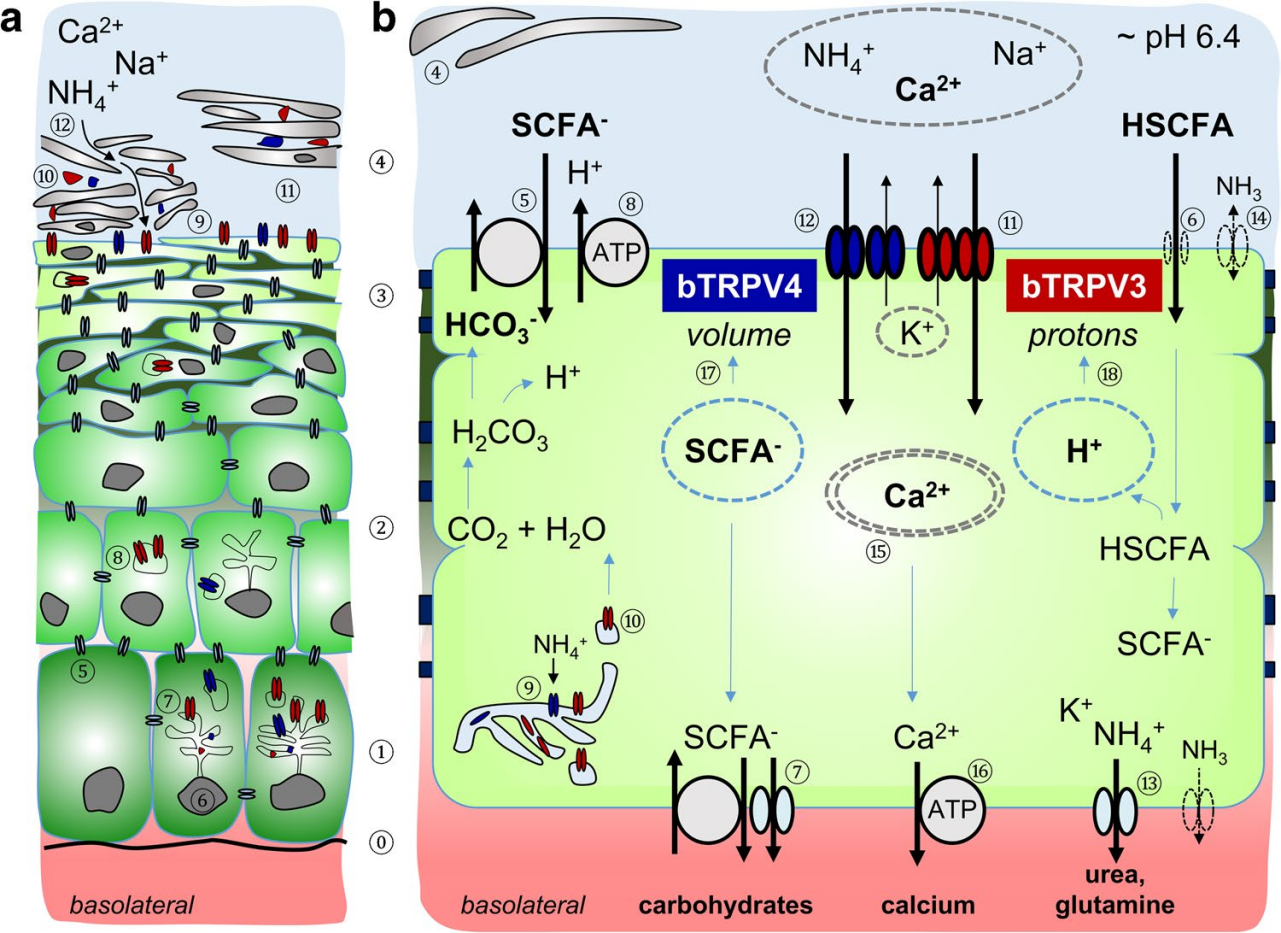
Model: bTRPV3 and bTRPV4 function in rumen. **a** Schematic representation showing the various layers of the ruminal epithelium. Starting from the basolateral side at the bottom, the basal lamina (⓪) serves as an attachment point for the cells of the *stratum basale* (①) which are the replicating cells of the epithelium. As cells grow upwards, they differentiate into cells of the *stratum spinosum* (②), *stratum granulosum* (③), and finally *stratum corneum* (④). Cells of the bottom three layers (①, ②, ③) are interconnected by gap junction proteins (⑤), forming a functional syncytium across which ions, molecules, and water can be transported. Tight junction proteins largely prevent paracellular transport. Cell nuclei (⑥) are in every cell of the *stratum basale* and *stratum spinosum*. Channel proteins are transcribed from mRNA within the endoplasmic reticulum (⑦). After assembly, the channels are incorporated into the lipid membrane of vesicles (⑧) for trafficking into the apical membrane of the *stratum granulosum* (⑨). Cells of the *stratum corneum* are surrounded by a protein envelope (shown in black) which displaces the lipid membrane (blue) and its now dysfunctional proteins (⑩). In parakeratosis, premature maturation leads to reinforced flattening of the cells of the *stratum granulosum*, often with loss of the endoplasmic reticulum and cell nuclei, while cells from the *stratum corneum* show residual cell nuclei and frequently detach from the underlying layers (⑪). In absorptive stratified epithelia, substrates can pass through the paracellular pathway between corneocytes to reach the apical membrane of the *stratum granulosum* (⑫). **b** Simplified transport model of the ruminal epithelium showing the functional syncytium between the *stratum basale* (①) *stratum spinosum* (②), and *stratum granulosum* (③). The *stratum corneum* is partially detached (④). SCFA from fermentation processes within the rumen supply energy to the animal and serve for carbohydrate anabolism. Apical uptake occurs as the anion (SCFA^−^) via transporters (⑤) or in the undissociated form (HSCFA, ⑥). Basolateral efflux involves anion channels or transporters (⑦). Dissociation of HSCFA leads to acidification with stimulation of pH regulatory mechanisms such as NHE and H^+^-ATPases (⑧). Following synthesis and assembly in the endoplasmic reticulum (⑨), some channels may be required for local functions (e.g. uptake of NH_4_^+^ for glutamine synthesis), while others are trafficked in vesicles (⑩) to the apical membrane of the *stratum granulosum*. After membrane insertion, bTRPV3 (red, ⑪) and bTRPV4 (blue, ⑫) mediate the apical uptake of cations including NH_4_^+^ and Ca^2+^. Basolateral efflux of NH_4_^+^ probably involves K^+^ channels (⑬). At the physiological pH of the rumen, transport of NH_3_ plays a minor role, with backflow possible if the NH_3_ gradient is inverted by a low luminal and a high cytosolic pH (⑭). After uptake (⑪, ⑫), Ca^2+^ is transported to the basolateral membrane bound to cytosolic buffers (⑮). Basolateral efflux may involve both Na^+^-dependent and Na^+^-independent mechanisms (⑯). When SCFA concentrations in the rumen are high, changes in cell volume (⑰) and cytosolic protons (⑱) open bTRPV4 (⑪) and bTRPV3 (⑫), respectively, leading to an increase in the transport of Ca^2+^ and NH_4_^+^ across the rumen as observed in vivo and in vitro. Functionally, the coupling of Ca^2+^ transport via TRP channels with proton extrusion may resemble Ca^2+^/H^+^ exchange

In rumen, mRNA and protein of TRPV3 was detected, and Ussing chamber studies using various different TRP agonists point towards a functional involvement of TRPV3 in this non-selective cation conductance [32, 56, 76, 78]. However, in addition to TRPV3, the bovine rumen also expresses mRNA for the non-selective cation channel TRPV4 [78]. A major goal of the current study was to sequence the bovine homologue (bTRPV4) and to investigate its functional expression and localization within the native ruminal epithelium. Furthermore, given the large quantities of ammonia (NH_4_^+^ or NH_3_) absorbed by the rumen in the cationic form (NH_4_^+^) [1, 3, 11], we wished to determine the permeability of bTRPV4 to NH_4_^+^. Since short-chain fatty acids (SCFA) stimulate the ruminal uptake of NH_4_^+^ [12, 13] and Ca^2+^ [44, 54, 81–83, 104, 110, 117] in vivo and in vitro, we finally investigated whether butyrate^−^ stimulates bTRPV3 and/or bTRPV4. Based on our data and literature, we present a model for the stimulation of ruminal cation transport by SCFA (Fig. 15).

### Expression of the bovine homologue of TRPV4 (bTRPV4) by the ruminal epithelium

Sequencing of bTRPV4 yielded a protein (QXI66840.1) with ~ 97% homology to that of humans (NP_067638.3), ~ 93% homology to the porcine TRPV4 (XM_013982949.2), and ~ 89% homology to that of the mouse (XM_006530432), all of which have an almost identical calculated molecular weight of ~ 98 kDa. Functionally important sites, such as the binding sites for protons [31, 112] or GSK1016790A [15], were conserved, as well as the pore region [108] which differed from that of TRPV3 by two amino acids (Supplement, Part G).

Two different antibodies (Thermo and ABIN) were established using overexpressing HEK-293 cells. In immunoblots, staining could be seen at ~ 130 kDa, reflecting the sum of bTRPV4 construct and its fused Strep and YFP tags (Fig. 1). Glycosylation is the most likely reason for the doubling of the band [87]. Control cells did not show staining. Likewise, in immunofluorescence imaging, only the overexpressing cells showed staining, which was localized in the cell membrane (Fig. 2). In immunoblots of protein from the bovine ruminal epithelium, a strong band emerged at ~ 60 kDa (Fig. 1). Since this band was observed using both established antibodies, it appears unlikely that it reflects non-specific binding. A more likely reason for the additional band is breakdown or splicing. In humans, many different splice variants of TRPV4 have been identified [4]. At higher protein concentrations and higher exposure times, a band emerged at ~ 100 kDa, reflecting the expected height for the untagged bTRPV4. The ~ 60 kDa band could again be detected, followed by bands of smaller molecular weight. In the mouse, a TRPV4 splice variant of ~ 66 kD has been identified (XP_036021298.1, 592 amino acids), possibly identical to an additional ~ 50 kDa band reported by the supplier of the Thermo antibody. In our study of the expression of TRPV4 by porcine gastrointestinal tissues [61], staining for TRPV4 was observed at a slightly lower level than expected. While this may reflect different splicing or breakdown, it should also be noted that in that study, we used a commercial gel with a higher polyacrylamide percentage (10%) instead of a freshly made gel with 7.5% as in the current study. Furthermore, in the current study, the duration of electrophoresis was longer. All of these factors influence the separation of proteins and the accuracy with which band height can be determined [35], so that differences should not be overinterpreted.

### Localization of bTRPV3 and bTRPV4 in the native bovine ruminal epithelium and in a cell culture model

Immunofluorescence staining was used for localization of bTRPV3 and bTRPV4 in the intact bovine ruminal epithelium (Fig. 3, 4). The apical staining pattern for bTRPV3 and bTRPV4 resembles that found in the (monolayered) porcine intestine [61, 102] and suggests a role in transport (See model in Fig. 15). Note that the cells of the *stratum corneum* are surrounded by a protein envelope, while the lipid membrane and its proteins are pushed out into the intercellular space. In the skin, secretion of further lipids seals the paracellular pathway to prevent loss of fluid [8]. Conversely, in mucous membranes such as the oral mucosa [115] or the rumen, the intercellular space of the *stratum corneum* remains permeable. The cells reaching from the *stratum basale* to the *stratum granulosum* are interconnected by gap junctions to form a functional syncytium [37]. Interestingly, keratinocytes grown in multiple layers on inserts showed an organizational structure that surprisingly resembled that of the native epithelium (Fig. 5, 6, and supplemental films). As in our previous study [96], the model expressed tight junction proteins with TEER values > 700 Ω · cm^2^.

In line with previous investigations of bovine rumen [53, 92], our preparations showed clear signs of parakeratosis. In cattle, this condition is induced by feeding diets rich in rapidly digestible carbohydrates with increased rates of fermentation, low ruminal pH, and high levels of SCFA and ammonia. This requires an adaption of the ruminal mucosa to allow a more rapid rate of transport [5]. Higher rates of mitosis are found in the *stratum basale.* Cells are pushed upwards into the *stratum spinosum* where they prematurely begin to form keratin within keratohyalin granules that are normally typical of the *stratum granulosum*. The granular cells within that layer prematurely lose cell organelles and cell nuclei [97], become very flat, and resemble cells of the *stratum corneum*, which conversely will frequently continue to express cell nuclei [92]. Furthermore, the *stratum corneum* may detach from the underlying layers [53, 97]. This may make it difficult to distinguish between cells of the two upper layers, but one of the most reliable properties of cells of the *stratum granulosum* persists, namely the expression of tight junction proteins, which are never found in the *stratum corneum* [97].

Judging from Figs. 3 and 4, in the ruminal epithelium, the boundary between the *stratum corneum* and the *stratum granulosum* is the point at which bTRPV3 and bTRPV4 are inserted into the membrane. Below this line, cells are tightly adjoined by properly inserted tight junctions [33, 97], while above, staining shows a disorganized pattern, reflecting the displacement of the lipid membrane and its proteins by the corneocyte envelope. It is possible to speculate that the enzymatic processes involved degrade not only the membrane lipids but also the proteins within, which might explain the high number of weak bands < 60 kDa in Fig. 1b and the dysmorphic staining of the *stratum corneum* in Fig. 3.

### Functional studies in Ussing chambers and using the patch-clamp technique

To test for functional expression of bTRPV4, the potent agonist GSK1016790A was used that is currently thought to be specific for TRPV4 [7]. Indeed, whole-cell experiments confirmed that at 50 nmol · L^−1^, GSK1016790A significantly stimulates Na^+^ and K^+^ currents in HEK-293 cells expressing bTRPV4, but not in bTRPV3 cells or controls (Tables 2 and 3, Figs. 8, 9, and 10). According to a recent study, the specific action of GSK1016790A on TRPV4 requires three binding sites [15], one of which (N474) is not conserved in TRPV3 (Supplement, Part G).

These experiments further show that the bTRPV4 conducts the NH_4_^+^ ion (Table 2 and Fig. 8 and 9). Given the high homology, this is also likely to be true for the human variant.

To test effects of GSK1016790A on native ruminal epithelia, Ussing chamber measurements were carried out with no electrochemical gradient present. In this situation, a current has to be energized by primary or secondary active transport. Accordingly, a rise in short-circuit current (*I*_sc_) will reflect an increase in transcellular transport—unless the transcellular conductance (*G*_t_) drops, which was not the case in any of the experiments (Fig. 7 and Table 1).

The concentration of GSK1016790A had to be raised to 2 µmol · L^−1^ before a response occurred. The response consisted of an initial increase in *I*_sc_ followed by a subsequent decrease. The *I*_sc_ rise is to be expected if GSK1016790A opens an apical non-selective cation channel. Influx of Na^+^ will depolarize the tissue, stimulating the efflux of K^+^ through the same pathway until the *I*_sc_ reverses sign, all as observed. The response strikingly resembled our observations for 2-APB (Fig. 7 and Table 1) and for menthol and thymol in previous studies [76, 78]. However, no effect on *G*_t_ was observed in contrast to what was seen with these agonists. It is tempting to speculate that the barrier-enhancing effects of bTRPV4 compensated for any increase in *G*_t_ [90].

Although non-specific effects cannot be ruled out, the simplest explanation is that GSK1016790A opened the non-selective bTRPV4 channel.

For comparison and to test the vitality of the tissues, 2-APB (500 µmol · L^−1^) was used. Unfortunately, there is currently no specific TRPV3 agonist, but based on patch-clamp and molecular biological data, a robust response was expected. While seen in isolation, a response to 2-APB certainly does not prove expression of bTRPV3, a failure to see a response would argue against functional expression of bTRPV3. Furthermore, 2-APB does not activate TRPV4 [46]. Note that expression of bTRPV3 by the rumen has been demonstrated on the level of the protein in a previous publication [56]. Furthermore, we have previously tested the effects of a number of less promiscuous TPRV3 agonists on the ruminal epithelium, but 2-APB was missing on our list [78].

Both the *I*_sc_ and the *G*_t_ response to 2-APB mirrored the previous observations after application of menthol or thymol [76, 78], arguing for a common pathway. As above, this response involved an initial increase in *I*_sc_, most likely reflecting influx of Na^+^, followed by a decrease caused by efflux of K^+^. A simultaneous increase in *G*_t_ may reflect both an opening of apical ion channels and an additional opening of the paracellular pathway, as discussed previously for other TRPV3 agonists [78]. Patch-clamp experiments on HEK-293 cells overexpressing bTRPV3 confirmed the stimulation of both Na^+^ and K^+^ conductances by a similar concentration of 2-APB (300 µmol · L^−1^) (Fig. 10, Table 3).

Mutagenesis analysis has shown that the effect of 2-APB on TRPV3 implicates two amino acid residues that are conserved when comparing the bovine to the human TRPV3 (Supplement, Part G) [46]. Apart from various other effects [43], 2-APB activates TRPV1, TRPV2, TRPV3, and TRPA1 and inhibits TRPC4, TRPC5, TRPC6, TRPM8, and TRPP1, while it has no effect on TRPV4, TRPV5, or TRPV6 [20, 21, 41, 46, 47]. As discussed, the rise in both *I*_sc_ and *G*_t_ argues against a channel block. Since no mRNA for either TRPV1 or TRPV2 was detected in the rumen [78], this leaves bTRPV3 and bTRPA1 as high ranking candidates for the effects of 2-APB.

It is certainly tempting to speculate that bTRPA1 is involved in the previously observed stimulatory effects of menthol on ruminal Ca^2+^ transport in Ussing chambers [32, 78]. Micromolar concentrations of menthol stimulate not only bTRPV3, but also bTRPA1 [49]. At a higher concentration > 0.05 mmol · L^−1^, however, menthol should inhibit bTRPA1 [49]. Conversely, ruminal tissues treated with 0.1 or 1 mmol · L^−1^ of menthol showed an *I*_sc_ and *G*_t_ response that mirrored the effects of 2-APB in the current study [76, 78]. Since bTRPM8 is not expressed by the rumen [32, 78], the response to menthol argues for an involvement of bTRPV3 that clearly exceeds any involvement of bTPRA1.

In this context, it should also be noted that while mRNA signals for TRPV3 and TRPV4 are robust, mRNA encoding for bTRPA1 was frequently weak [32] or even near the limit of detection [76]. Possibly, expression levels of TRPA1 are low because its role is primarily in signalling, rather than in bulk transport of cations. In the gut, the activation of TRPA1 by a plethora of membrane-permeable substances may help with the detection of noxious substances [49]. In the colon, activation of TRPA1 induced EP4-mediated signalling with anion secretion [48, 61]. A role for TRPA1 in signalling is also suggested by the fact that gain-of-function mutations of TRPA1 cause hereditary pain syndromes with no signs of dermal disease [49]. However, we do not exclude participation in cation transport [61], and further investigations are clearly required.

### Stimulation of Na^+^ and Ca^2+^ transport by butyrate^−^

Our interest was triggered by the fact that SCFA, in general, and butyrate^−^, in particular, stimulate Ca^2+^ uptake by the rumen [44, 54, 81–83, 104, 110, 117] and by the colon of rats [59] and humans [100], all of which express TRPV3 and TRPV4. Since in the rumen, effects of butyrate^−^ have been shown to be stronger than those of acetate^−^ or propionate^−^ [82], we tested the effects of this SCFA on HEK-293 cells expressing bTRPV3 or bTRPV4 and control cells via the whole-cell configuration of the patch-clamp technique and via Ca^2+^ imaging.

In monogastric species, it has been solidly established that the major site of Ca^2+^ absorption is the duodenum and the upper jejunum, where the bulk of Ca^2+^ is apically taken up via TRPV6, crosses the cytosol bound to calbindin-D_9K_, and is basolaterally extruded via a Ca^2+^-ATPase (PMCA1b), all under the control of calcitriol [42, 80]. Conversely, in cattle and sheep, it has been estimated that roughly 50% of total Ca^2+^ absorption takes place pre-intestinally [80], although these amounts may vary [118]. Ruminal uptake is almost exclusively transcellular and energized via basolateral Na^+^/Ca^2+^ exchange [44, 55, 82] and possibly also PMCA1 [83]. Expression of calbindin-D_9K_ was detected in goat rumen [88] but not in the rumen of sheep [116, 117]. Although apical uptake of Ca^2+^ involves an electrogenic component [44, 54, 118], no mRNA for the epithelial calcium channels TRPV5 and TRPV6 was detected [32, 78, 83, 116, 117]. Furthermore, and despite considerable interest by researchers and farmers alike, there is no sign of a stimulation of ruminal Ca^2+^ absorption by calcitriol [117, 118] or vitamin D [88].

The protonated form of butyrate^−^ (butyric acid) is a weak acid with a pK_a_ ~ 4.8. At pH 7.4, 0.25% is present in butyric acid, rising to 2.45% at pH 6.4. In the classical model, the uncharged butyric acid diffuses into the cytosol, where it dissociates, releasing butyrate^−^ and a proton [95] (Fig. 15). Since effects of SCFA are higher at low pH, protons are likely a key factor for the stimulation of Ca^2+^ transport. In similar fashion, elevation of mucosal chloride–which acidifies the cytosol via stimulation of Cl^−^/HCO_3_^−^ exchange–enhances ruminal Ca^2+^ transport [54]. In that study, it could also be shown that both electrogenic and electroneutral mechanisms are involved in proton-sensitive Ca^2+^ transport. Furthermore, acidification of the cytosol by application of amiloride blocks extrusion of protons via Na^+^/H^+^ exchange (NHE) and stimulates Ca^2+^ transport. These findings led to the postulation of a Ca^2+^/H^+^ exchanger [59, 82], the identity of which continues to remain unclear. The model was recently challenged by a study in which NHE was blocked by replacing Na^+^ with NMDG^+^, leading to a drop in net Ca^2+^ flux [32]. In various members of the TRP family including TRPV3, NMDG^+^ can enter the pore in response to triggers that include membrane stretch, interfering with the passage of other cations [28, 79]. The blocking effects of NMDG^+^ suggest that the stimulatory effects of SCFA on ruminal Ca^2+^ transport may involve TRP channels. The whole-cell patch-clamp data suggest that intracellular protons activate bTRPV3 (Table 4 and Fig. 12). Capacitance measurements in these experiments suggest that volume-related effects were of minor importance, which may explain the failure of butyrate^−^ to significantly stimulate bTRPV4. However, an involvement of volume changes in the responses of individual cells overexpressing the highly stretch-sensitive bTRPV4 appears possible [68] (Fig. 11).

Our finding that TRPV3, but not TRPV4, is opened by intracellular protons is not new [17, 31]. Single-channel data suggest that of the four channels TRPV1, TRPV2, TRPV3, and TRPV4, only TRPV3 is directly activated by intracellular protons. Mutagenesis analysis showed that key cytoplasmic residues of the TRPV3 channel are required [17, 31, 112] (Supplement, Part G). Conversely, extracellular protons do not activate the channels, although they may augment the activity of agonists [17, 89].

In light of these deliberations, it is somewhat surprising that in the calcium imaging experiments, all three groups of cells showed a significant and reversible increase in [Ca^2+^]_i_ due to NaBu 6.4. However, effects were significantly higher in the cells overexpressing bTRPV3 or bTRPV4. The reason for the butyrate^−^-induced rise of [Ca^2+^]_i_ in the control cells may simply reflect the classical release of Ca^2+^ from intracellular buffers after binding of H^+^ [66]. Proton-induced opening of bTRPV3 is a plausible explanation for the high rise in [Ca^2+^]_i_ observed in the bTRPV3 expressing cells [17, 31]. The peak and subsequent drop possibly reflect negative feedback after binding of Ca^2+^ to a calmodulin binding site in the ankyrin repeat domain [74, 113].

The positive [Ca^2+^]_i_ response of the bTRPV4 cells to NaBu 6.4 was not expected. However, while in the whole-cell experiments, changes in volume were largely prevented by backflow into the pipette, this outlet was not available to the intact, fura-2–loaded cells. Influx of butyrate^−^ should lead to swelling–which can be expected to activate the volume sensor TRPV4 [68].

### TRPV3 and TRPV4: team players in ruminal cation transport?

The functional expression of TRPV4 by keratinocytes is well-documented, notably also in the oesophageal epithelium [87, 101]. The current study clearly confirms expression of TRPV4 by the ruminal epithelium, which evolved from the oesophagus. However, both the relatively weak band for the full-length protein at ~ 100 kDa in the immunoblots and the high concentrations of GSK1016790A required to observe a functional response in Ussing chambers suggest that functional expression of bTRPV4 by the rumen may be lower than that of bTRPV3. In this context, it is interesting to note that neither a gain- nor a loss-of-function of TRPV4 causes skin disease, although pathologies of skeletal growth and neuropathy are observed [10, 99]. Conversely, gain-of-function mutations of TRPV3 lead to mutilating hyperkeratosis in humans [26, 69]. Since loss-of-function mutations of TRPV3 have a mild phenotype [70], it is possible to speculate that TRPV4 and/or other channels are upregulated for compensation.

Likewise, in the rumen, the relative importance of TRPV4 in ensuring an adequate efflux of cations from the rumen might rise under certain conditions–such as high ruminal osmolarity. As mentioned, TRPV4 channels are outstanding volume sensors (e.g. after a hypotonic challenge) [99]. Furthermore, a role in the formation of intercellular junctions between keratinocytes is discussed [10, 90]. Both functions might be involved in the impressive and reversible changes in ruminal barrier function observed in response to hyperosmotic challenges [58]. Maybe TRPV3 and TRPV4 are team players—with TRPV3 doing most of the work under normal circumstances and TRPV4 stepping in when it is necessary.

In conclusion, we present evidence for the expression of bTRPV4 by the apical membrane of the *stratum granulosum* of the ruminal epithelium, with cytosolic staining in the epithelial layers below (Fig. 15a). Based on the weak bands for the full-length protein and the high amounts of GSK1016790A required to stimulate the channel in the native epithelium, its functional role in cation transport across the rumen may lag behind that of bTRPV3. Stimulation of ruminal Ca^2+^ transport by SCFA may primarily involve bTRPV3, which is opened by cytosolic protons (Fig. 15b). Activation of bTRPV4 after membrane stretch may contribute. In conjunction with the apically expressed H^+^-ATPase [52], a functionally coupled exchange of protons with cations including Ca^2+^ and Na^+^ can be expected to occur [59, 82]. This mechanism might also explain functional exchange of Na^+^ with protons in situations where mucosal pH is very low with an insufficient driving force for NHE.

When SCFA concentrations become excessive or the ruminal pH drops low, as in cattle fed high-soluble carbohydrate, ruminal hyperkeratosis and parakeratosis may develop [5, 6, 92]. The histological changes show similarities to those observed in humans suffering from a gain-of-function mutation of TRPV3 [69]. Future work will have to determine whether or not influx of NH_4_^+^ has a role to play in these changes [57]. Furthermore, more work is necessary to determine the quantitative roles of TRPV3 and TRPV4 in mediating transport of NH_4_^+^ and Ca^2+^, respectively. This will require more specific agonists and antagonists. A greater understanding of the precise mechanisms involved and tools suitable for selectively modulating them may help ruminants and humans alike.

## Supplementary information

ESM 1 (PDF 333 kb)

ESM 2 (MP4 7.57 mb)

ESM 3 (MP4 2.22 mb)

## Data Availability

All data generated and/or analysed during the current study are available from the corresponding author on reasonable request.

## Code availability

Available on reasonable request from F.S.
