# Supplementary material for: Effects of butyrate− on ruminal Ca2+ transport: evidence for the involvement of apically expressed TRPV3 and TRPV4 channels
Source: Pflugers Arch. 2022 Jan 31;474(3):315–42. doi: 10.1007/s00424-021-02647-7 (PMC8837523; doi:10.1007/s00424-021-02647-7)
Supplement: Supplementary file 1 — (PDF 333 kb) [file 424_2021_2647_MOESM1_ESM.pdf]

<u>**Supplementary Material**</u>

Effects of butyrate$^-$ on ruminal Ca^{2+} transport: evidence for the involvement of apically expressed TRPV3 and TRPV4 channels

Pflügers Archiv – European Journal of Physiology

Franziska Liebe[1], Hendrik Liebe[1,2], Stefan Mergler[3], Gerhard Sponder[1], Friederike Stumpff[1,4]

email: stumpff@zedat.fu-berlin.de
[1]Institute of Veterinary Physiology, Freie Universität Berlin, Berlin, Germany
[2]Department of Biology, Chemistry, and Pharmacy, Freie Universität Berlin, Germany
[3]Institute of Experimental Ophthalmology, Charité-Universitätsmedizin Berlin, Germany
[4]Institute of Physiology, Health and Medical University, Potsdam, Germany

Content

A) Ruminal cell culture 2

B) p5TO-bTRPV4-Strep-YFP vector 3

C) Immunoblotting 5

D) Modulators and solutions for patch-clamping and calcium imaging 6

E) Whole-cell patch-clamp experiments 7

F) Data analysis 8

G) Alignment of the human and bovine homologues of TRPV3 and TRPV4 9

A) Ruminal cell culture

Papillae were cut from the ruminal tissue and repeatedly washed in PBS without Ca^{2+} and Mg^{2+} (4 % penicillin and streptomycin), all as described previously [4,15]. The papillae were agitated in a trypsin-EDTA-solution (2.5 g · L^{-1} porcine trypsin and 0.2 g · L^{-1} EDTA) at 37 °C. After 30 and 60 minutes, the suspension was discarded, while the papillae were agitated again in fresh trypsin-EDTA-solution. This procedure was repeated to gain the following fractions 3, 4, and 5 which were filtered through gauze and centrifuged three times (4 min, 200 g, 23 °C) with removal of supernatant and resuspension in 10 mL PBS with Ca^{2+} and Mg^{2+} (1 % penicillin and streptomycin). Finally, $5 \cdot 10^5$ cells were seeded in medium M1 (M199 with Earle's salts, with stable L-glutamine and 2.2 g · L^{-1} $NaHCO_3$, #31150022, Thermo Fisher Scientific, Waltham, MA, USA) containing 0.11 g · L^{-1} sodium pyruvate, 15 % FBS (foetal bovine serum), 1 % penicillin and streptomycin, 50 mg · L^{-1} gentamycin, $2.4 \cdot 10^5$ U · L^{-1} nystatin, and 100 mg · L^{-1} kanamycin into cell culture dishes (Ø 60 mm) coated with collagen A (Biochrom). After two days in culture, cells were washed thrice in PBS without Ca^{2+} and Mg^{2+} and cultivation continued in medium M2 (Dulbecco's MEM with 4500 mg · L^{-1} glucose, stable L-glutamine, 0.11 g · L^{-1} sodium pyruvate, and 2.2 g · L^{-1} $NaHCO_3$, D0822) containing 10 % FBS, 1 % penicillin and streptomycin, 5 ng · mL^{-1} epidermal growth factor, and 1 % Insulin/Transferrin/Selenite (Thermo Fisher Scientific).

For immunofluorescence staining, ~ $5 \cdot 10^5$ ruminal cells · mL^{-1} were seeded directly onto cell culture inserts (Ø 12 mm, PCF membrane, pore size 0.4 µm, Merck Millipore, Darmstadt, Germany) and cultivated with medium M2 for about 20 days. Before staining, transepithelial electrical resistance (TEER) was monitored (Epithelial Volt/Ohm (TEER) Meter, World Precision Instruments, Hertfordshire, UK). Values were corrected for the resistance of control inserts.

B) p5TO-*bTRPV4-Strep-YFP* vector

The entire vector contains 9017 base pairs. Ligated into the p5TO vector, the *bTRPV4* coding sequence (2613 base pairs) is highlighted in green, followed by *Strep* tag (blue) with a short bridge structure to *YFP* (yellow fluorescent protein, yellow).

GACGGATCGGGAGATCTCCCGATCCCCTATGGTGCACTCTCAGTACAATCTGCTCTGATGCCGCATAGTTAAGCCAGTATC
TGCTCCCTGCTTGTGTGTTGGAGGTCGCTGAGTAGTGCGCGAGCAAAATTTAAGCTACAACAAGGCAAGGCTTGACCGACA
ATTGCATGAAGAATCTGCTTAGGGTTAGGCGTTTTGCGCTGCTTCGCGATGTACGGGCCAGATATACGCGTTGACATTGAT
TATTGACTAGTTATTAATAGTAATCAATTACGGGGTCATTAGTTCATAGCCCATATATGGAGTTCCGCGTTACATAACTTACG
GTAAATGGCCCGCCTGGCTGACCGCCCAACGACCCCCGCCCATTGACGTCAATAATGACGTATGTTCCCATAGTAACGCCA
ATAGGGACTTTCCATTGACGTCAATGGGTGGAGTATTTACGGTAAACTGCCCACTTGGCAGTACATCAAGTGTATCATATGC
CAAGTACGCCCCCTATTGACGTCAATGACGGTAAATGGCCCGCCTGGCATTATGCCCAGTACATGACCTTATGGGACTTTC
CTACTTGGCAGTACATCTACGTATTAGTCATCGCTATTACCATGGTGATGCGGTTTTGGCAGTACATCAATGGGCGTGGATA
GCGGTTTGACTCACGGGGATTTCCAAGTCTCCACCCCATTGACGTCAATGGGAGTTTGTTTTGGCACCAAAATCAACGGGA
CTTTCCAAAATGTCGTAACAACTCCGCCCCATTGACGCAAATGGGCGGTAGGCGTGTACGGTGGGAGGTCTATATAAGCAG
AGCTCTCCCTATCAGTGATAGAGATCTCCCTATCAGTGATAGAGATCGTCGACGAGCTCGTTTAGTGAACCGTCAGATCGC
CTGGAGACGCCATCCACGCTGTTTTGACCTCCATAGAAGACACCGGGACCGATCCAGCCTCCGGACTCTAGCGTTTAAACT
TCACC**ATG**GCGGATCCTGGCGAAGGCCCTCGTGTGGGGCCAGGGGAGATGGCCGAGTCGCCCGGGGATGAGAGTGGGC
CCCCCGGCGGGGAGGCCTTCCCCCTGTCATCACTGGCCAACCTGTTCGAGGGGGAAGACGGCTCCCCGTCGCCCTCGCC
AGCTGATGGCGGTCGCTCCACCGGCCCAGGCGACGGGCGACCCAACCTGCGCATGAAGTTCCAGGGCGCCTTCCGCAAG
GGGGTGCCCAACCCCATCGACCTGCTGGAGTCCACCCTGTACGAGTCCTCCGTGGTGCCCGGGCCCAAGAAGGCGCCCA
TGGACTCGCTGTTTGACTACGGCACCTATCGTCACCACCCCAGTGACAACAAGCGGTGGAGGAGGAAGGTCATAGAGAAG
CAGCCACAGAGCCCCAAAGCTCCCGCACCCCAGCCGCCTCCCATCCTCAAAGTCTTCAACCGGCCTATCCTCTTTGACATC
GTGTCCCGGGGCTCCACAGCTGACCTGGATGGGCTGCTCCCCTTCTTGCTGACCCACAAGAAGCGCCTCACGGACGAGG
AGTTCCGGGAACCGTCCACCGGGAAGACCTGCCTGCCCAAGGCCCTGCTGAATCTGAGCAACGGCCGCAATGACACCATC
CCTGTGCTTCTGGACATCGCCGAGCGCACGGGCAACATGCGGGAATTCATCAACTCGCCCTTCCGAGACATCTACTACCG
AGGCCAGACCGCCCTGCACATCGCCATCGAACGCCGCTGCAAACACTATGTGGAGCTGCTGGTGGCCCAAGGAGCCGAC
GTCCATGCCCAGGCGCGGGGCCGCTTCTTCCAGCCCAAGGACGAGGGCGGCTACTTCTACTTCGGTGAGCTGCCCCTGT
CGCTGGCTGCCTGCACCAACCAGCCCCACATCGTCAACTACCTGACGGAGAACCCGCACAAGAAGGCGGACATGCGGCG
CCAGGACTCGCGCGGCAACACGGTGCTGCACGCGCTGGTGGCCATTGCCGACAACACCCGCGAGAACACCAAGTTCGTC
ACCAAGATGTACGACCTGCTGCTGCTCAAGTGTGCCCGCCTCTTCCCCGACAGCAACCTGGAGGCCGTGCTCAACAATGA
CGGCCTCTCGCCCCTCATGATGGCCGCCAAGACGGGCAAGATTGGGATCTTCCAACACATCATCCGTCGGGAGGTGACAG
ACGAGGACACCAGGCACCTGTCGCGCAAGTTCAAGGACTGGGCCTATGGGCCGGTGTACTCCTCGCTCTACGACCTCTCC
TCCCTGGACACATGTGGGGAGGAGACCTCTGTGCTGGAGATCCTGGTGTACAACAGCAAGATCGAGAACCGCCACGAGAT
GCTGGCCGTGGAACCCATCAACGAACTACTGCGGGACAAGTGGCGGAAGTTCGGGGCTGTCTCCTTCTACATCAACGTGG
TCTCCTATCTGTGTGCCATGGTCATCTTTACCCTCACCGCCTACTACCAGCCTCTCGAGGGCACTCCGCCATACCCTTACC
GCACCACAATGGACTACCTGAGGCTGGCCGGCGAGATCATCACACTCTTCACTGGGATCCTGTTCTTTTTCACCAACATCA
AAGACTTGTTCATGAAGAAATGCCCTGGAGTGAACTCTCTTTTCATCGACGGCTCCTTCCAGCTACTCTACTTCATCTACTCT
GTGCTGGTGATTGTCTCAGCGGCCCTCTACCTGGCGGGGATCGAGGCGTACCTGGCCGTCATGGTCTTTGCCTTGGTCCT
GGGCTGGATGAACGCCCTTTACTTCACCCGCGGGCTGAAGCTGACGGGGACCTATAGCATCATGATCCAGAAGATCCTCT
TCAAAGACCTTTTCCGCTTCCTGCTGGTCTACTTGCTCTTCATGATCGGCTACGCGTCAGCCCTGGTCTCGCTCCTGAACC
CGTGTGCCAACTTGAAGGTGTGCGATGAGGACCACGCCAACTGCACAGTGCCCACCTACCCCTCATGCCGCGACAGCGAG
ACCTTCAGCACCTTCCTCCTGGACCTCTTCAAGCTCACCATCGGCATGGGCGACCTGGAGATGCTGAGCAGCACCAAGTA
CCCCGTGGTCTTCATCATCCTGCTGGTCACGTACATCATCCTCACATTCGTGCTGCTCCTTAACATGCTCATCGCCCTCATG
GGGGAGACAGTGGGCCAGGTGTCCAAGGAGAGCAAGCACATCTGGAAGCTGCAGTGGGCCACCACCATCCTGGACATCG
AGCGCTCCTTCCCCGTGTTCCTGAGGAAGGCCTTCCGCTCCGGTGAAATGGTGACCGTGGGCAAGAGCTCGGACGGCAC
TCCAGACCGCAGGTGGTGCTTCAGGGTGGACGAGGTGAACTGGTCTCACTGGAACCAGAACTTGGGCATCATTAACGAGG
ACCCCGGCAAGAACGAGAACTACCAGTACTATGGCTTCTCGCACACCGTGGGCCGCCTCCGGAGGGATCGCTGGTCCTCA
GTGGTGCCCCGCGTGGTAGAGCTCAACAAGAACTCCAACCCGGACGAGGTGGTGGTGCCTCTGGACAACGTAGGGAACC
CCAGCTGCGACGGCCACCAGCAGAGTTACCCCCCTAAGTGGAGGACTGACGACGCCCCGCTCTCGGCCTGGAGCCACCC
GCAGTTCGAGAAAGGTGGAGGTTCCGGAGGTGGATCGGGAGGTTCGGCCTGGTCGCACCCGCAGTTCGAAAAAGCGGCC
GCGGTCGACGTGAGCAAGGGCGAGGAGCTGTTCACCGGGGTGGTGCCCATCCTGGTCGAGCTGGACGGCGACGTAAAC
GGCCACAAGTTCAGCGTGTCCGGCGAGGGCGAGGGCGATGCCACCTACGGCAAGCTGACCCTGAAGTTCATCTGCACCA
CCGGCAAGCTGCCCGTGCCCTGGCCCACCCTCGTGACCACCTTCGGCTACGGCCTGCAGTGCTTCGCCCGCTACCCCGA
CCACATGAAGCAGCACGACTTCTTCAAGTCCGCCATGCCCGAAGGCTACGTCCAGGAGCGCACCATCTTCTTCAAGGACG
ACGGCAACTACAAGACCCGCGCCGAGGTGAAGTTCGAGGGCGACACCCTGGTGAACCGCATCGAGCTGAAGGGCATCGA
CTTCAAGGAGGACGGCAACATCCTGGGGCACAAGCTGGAGTACAACTACAACAGCCACAACGTCTATATCATGGCCGACA
AGCAGAAGAACGGCATCAAGGTGAACTTCAAGATCCGCCACAACATCGAGGACGGCAGCGTGCAGCTCGCCGACCACTAC
CAGCAGAACACCCCCATCGGCGACGGCCCCGTGCTGCTGCCCGACAACCACTACCTGAGCTACCAGTCCGCCCTGAGCA
AAGACCCCAACGAGAAGCGCGATCACATGGTCCTGCTGGAGTTCGTGACCGCCGCCGGGATCACTCTCGGCATGGACGA
GCTGTACAAGGGGCCCGTTTAAACCCGCTGATCAGCCTCGACTGTGCCTTCTAGTTGCCAGCCATCTGTTGTTTGCCCCTC
CCCCGTGCCTTCCTTGACCCTGGAAGGTGCCACTCCCACTGTCCTTTCCTAATAAAATGAGGAAATTGCATCGCATTGTCTG
AGTAGGTGTCATTCTATTCTGGGGGGTGGGGTGGGGCAGGACAGCAAGGGGGAGGATTGGGAAGACAATAGCAGGCATG

CTGGGGATGCGGTGGGCTCTATGGCTTCTGAGGCGGAAAGAACCAGCTGGGGCTCTAGGGGGTATCCCCACGCGCCCTG
TAGCGGCGCATTAAGCGCGGCGGGTGTGGTGGTTACGCGCAGCGTGACCGCTACACTTGCCAGCGCCCTAGCGCCCGCT
CCTTTCGCTTTCTTCCCTTCCTTTCTCGCCACGTTCGCCGGCTTTCCCCGTCAAGCTCTAAATCGGGGGCTCCCTTTAGGGT
TCCGATTTAGTGCTTTACGGCACCTCGACCCCAAAAAACTTGATTAGGGTGATGGTTCACGTAGTGGGCCATCGCCCTGAT
AGACGGTTTTTCGCCCTTTGACGTTGGAGTCCACGTTCTTTAATAGTGGACTCTTGTTCCAAACTGGAACAACACTCAACCC
TATCTCGGTCTATTCTTTTGATTTATAAGGGATTTTGCCGATTTCGGCCTATTGGTTAAAAAATGAGCTGATTTAACAAAAATT
TAACGCGAATTAATTCTGTGGAATGTGTGTCAGTTAGGGTGTGGAAAGTCCCCAGGCTCCCCAGCAGGCAGAAGTATGCAA
AGCATGCATCTCAATTAGTCAGCAACCAGGTGTGGAAAGTCCCCAGGCTCCCCAGCAGGCAGAAGTATGCAAAGCATGCAT
CTCAATTAGTCAGCAACCATAGTCCCGCCCCTAACTCCGCCCATCCCGCCCCTAACTCCGCCCAGTTCCGCCCATTCTCCG
CCCCATGGCTGACTAATTTTTTTTATTTATGCAGAGGCCGAGGCCGCCTCTGCCTCTGAGCTATTCCAGAAGTAGTGAGGA
GGCTTTTTTGGAGGCCTAGGCTTTTGCAAAAAGCTCCCGGGAGCTTGTATATCCATTTTCGGATCTGATCAGCACGTGATGA
AAAAGCCTGAACTCACCGCGACGTCTGTCGAGAAGTTTCTGATCGAAAAGTTCGACAGCGTCTCCGACCTGATGCAGCTCT
CGGAGGGCGAAGAATCTCGTGCTTTCAGCTTCGATGTAGGAGGGCGTGGATATGTCCTGCGGGTAAATAGCTGCGCCGAT
GGTTTCTACAAAGATCGTTATGTTTATCGGCACTTTGCATCGGCCGCGCTCCCGATTCCGGAAGTGCTTGACATTGGGGAA
TTCAGCGAGAGCCTGACCTATTGCATCTCCCGCCGTGCACAGGGTGTCACGTTGCAAGACCTGCCTGAAACCGAACTGCC
CGCTGTTCTGCAGCCGGTCGCGGAGGCCATGGATGCGATCGCTGCGGCCGATCTTAGCCAGACGAGCGGGTTCGGCCCA
TTCGGACCGCAAGGAATCGGTCAATACACTACATGGCGTGATTTCATATGCGCGATTGCTGATCCCCATGTGTATCACTGG
CAAACTGTGATGGACGACACCGTCAGTGCGTCCGTCGCGCAGGCTCTCGATGAGCTGATGCTTTGGGCCGAGGACTGCCC
CGAAGTCCGGCACCTCGTGCACGCGGATTTCGGCTCCAACAATGTCCTGACGGACAATGGCCGCATAACAGCGGTCATTG
ACTGGAGCGAGGCGATGTTCGGGGATTCCCAATACGAGGTCGCCAACATCTTCTTCTGGAGGCCGTGGTTGGCTTGTATG
GAGCAGCAGACGCGCTACTTCGAGCGGAGGCATCCGGAGCTTGCAGGATCGCCGCGGCTCCGGGCGTATATGCTCCGCA
TTGGTCTTGACCAACTCTATCAGAGCTTGGTTGACGGCAATTTCGATGATGCAGCTTGGGCGCAGGGTCGATGCGACGCAA
TCGTCCGATCCGGAGCCGGGACTGTCGGGCGTACACAAATCGCCCGCAGAAGCGCGGCCGTCTGGACCGATGGCTGTGT
AGAAGTACTCGCCGATAGTGGAAACCGACGCCCCAGCACTCGTCCGAGGGCAAAGGAATAGCACGTGCTACGAGATTTCG
ATTCCACCGCCGCCTTCTATGAAAGGTTGGGCTTCGGAATCGTTTTCCGGGACGCCGGCTGGATGATCCTCCAGCGCGGG
GATCTCATGCTGGAGTTCTTCGCCCACCCCAACTTGTTTATTGCAGCTTATAATGGTTACAAATAAAGCAATAGCATCACAAA
TTTCACAAATAAAGCATTTTTTTCACTGCATTCTAGTTGTGGTTTGTCCAAACTCATCAATGTATCTTATCATGTCTGTATACC
GTCGACCTCTAGCTAGAGCTTGGCGTAATCATGGTCATAGCTGTTTCCTGTGTGAAATTGTTATCCGCTCACAATTCCACAC
AACATACGAGCCGGAAGCATAAAGTGTAAAGCCTGGGGTGCCTAATGAGTGAGCTAACTCACATTAATTGCGTTGCGCTCA
CTGCCCGCTTTCCAGTCGGGAAACCTGTCGTGCCAGCTGCATTAATGAATCGGCCAACGCGCGGGGAGAGGCGGTTTGC
GTATTGGGCGCTCTTCCGCTTCCTCGCTCACTGACTCGCTGCGCTCGGTCGTTCGGCTGCGGCGAGCGGTATCAGCTCAC
TCAAAGGCGGTAATACGGTTATCCACAGAATCAGGGGATAACGCAGGAAAGAACATGTGAGCAAAAGGCCAGCAAAAGGC
CAGGAACCGTAAAAAGGCCGCGTTGCTGGCGTTTTTCCATAGGCTCCGCCCCCCTGACGAGCATCACAAAAATCGACGCT
CAAGTCAGAGGTGGCGAAACCCGACAGGACTATAAAGATACCAGGCGTTTCCCCCTGGAAGCTCCCTCGTGCGCTCTCCT
GTTCCGACCCTGCCGCTTACCGGATACCTGTCCGCCTTTCTCCCTTCGGGAAGCGTGGCGCTTTCTCATAGCTCACGCTGT
AGGTATCTCAGTTCGGTGTAGGTCGTTCGCTCCAAGCTGGGCTGTGTGCACGAACCCCCCGTTCAGCCCGACCGCTGCGC
CTTATCCGGTAACTATCGTCTTGAGTCCAACCCGGTAAGACACGACTTATCGCCACTGGCAGCAGCCACTGGTAACAGGAT
TAGCAGAGCGAGGTATGTAGGCGGTGCTACAGAGTTCTTGAAGTGGTGGCCTAACTACGGCTACACTAGAAGAACAGTATT
TGGTATCTGCGCTCTGCTGAAGCCAGTTACCTTCGGAAAAAGAGTTGGTAGCTCTTGATCCGGCAAACAAACCACCGCTGG
TAGCGGTTGGTTTTTTTGTTTGCAAGCAGCAGATTACGCGCAGAAAAAAAGGATCTCAAGAAGATCCTTTGATCTTTTCTACG
GGGTCTGACGCTCAGTGGAACGAAAACTCACGTTAAGGGATTTTGGTCATGAGATTATCAAAAAGGATCTTCACCTAGATCC
TTTTAAATTAAAAATGAAGTTTTAAATCAATCTAAAGTATATATGAGTAAACTTGGTCTGACAGTTACCAATGCTTAATCAGTG
AGGCACCTATCTCAGCGATCTGTCTATTTCGTTCATCCATAGTTGCCTGACTCCCCGTCGTGTAGATAACTACGATACGGGA
GGGCTTACCATCTGGCCCCAGTGCTGCAATGATACCGCGAGACCCACGCTCACCGGCTCCAGATTTATCAGCAATAAACCA
GCCAGCCGGAAGGGCCGAGCGCAGAAGTGGTCCTGCAACTTTATCCGCCTCCATCCAGTCTATTAATTGTTGCCGGGAAG
CTAGAGTAAGTAGTTCGCCAGTTAATAGTTTGCGCAACGTTGTTGCCATTGCTACAGGCATCGTGGTGTCACGCTCGTCGTT
TGGTATGGCTTCATTCAGCTCCGGTTCCCAACGATCAAGGCGAGTTACATGATCCCCCATGTTGTGCAAAAAAGCGGTTAG
CTCCTTCGGTCCTCCGATCGTTGTCAGAAGTAAGTTGGCCGCAGTGTTATCACTCATGGTTATGGCAGCACTGCATAATTCT
CTTACTGTCATGCCATCCGTAAGATGCTTTTCTGTGACTGGTGAGTACTCAACCAAGTCATTCTGAGAATAGTGTATGCGGC
GACCGAGTTGCTCTTGCCCGGCGTCAATACGGGATAATACCGCGCCACATAGCAGAACTTTAAAAGTGCTCATCATTGGAA
AACGTTCTTCGGGGCGAAAACTCTCAAGGATCTTACCGCTGTTGAGATCCAGTTCGATGTAACCCACTCGTGCACCCAACT
GATCTTCAGCATCTTTTACTTTCACCAGCGTTTCTGGGTGAGCAAAAACAGGAAGGCAAAATGCCGCAAAAAAGGGAATAAG
GGCGACACGGAAATGTTGAATACTCATACTCTTCCTTTTTCAATATTATTGAAGCATTTATCAGGGTTATTGTCTCATGAGCG
GATACATATTTGA ATGTATTTAGAAAAATAAACAAATAGGGGTTCCGCGCACATTTCCCCGAAAAGTGCCACCTGACGTC

C) Immunoblotting

For protein extraction, solvents and samples were cooled throughout the experiments to minimize protein degradation.

For bovine rumen, RIPA buffer (500 µL; in mmol L^{-1}: 25 HEPES, 2 EDTA, 25 NaF, protease inhibitor (cOmplete™, mini, Roche, Basel, Switzerland), 1% sodium dodecyl sulfate (SDS)) was added to the defrosted tissue (200 mg) together with two metal beads. The tissue was homogenized 30 times in a mixer mill for 2 min with alternate 2 min cooling in ice bath (MM200, RetschGmbH, Haan, Germany), followed by a clarifying spin (15 min, 20 000 g, 4 °C). The supernatant containing the protein was transferred into a new tube.

For HEK-293 cells, after washing with phosphate-buffered saline (PBS), cells were harvested mechanically by scraping in PBS. After centrifugation (500 g, 5 min), the cell pellet was resuspended in PBS (1 mL) and transferred into a new tube. PBS was removed via centrifugation (700 g, 4 min). The cell pellet was lysed in RIPA buffer (100 µL) for 30 min with intermediate gentle agitation. Finally, tube was put in an ultrasound bath (5 min) and centrifuged by a clarifying spin (20 min, 15 000 g, 4 °C). The supernatant contained the protein.

All protein samples were stored at -80 °C. Concentrations were determined prior to the experiment using a Pierce™ 660 nm protein assay kit (Thermo Fisher Scientific, Waltham, MA, USA). All samples were denatured in Laemmli sample buffer (2x; #1610737, Bio-Rad Laboratories GmbH, Munich, Germany) supplemented with 2-Mercaptoethanol (5 vol%) and electrophoresed on freshly prepared polyacrylamide-gels (7.5 %, SDS-PAGE) in Tris-Glycine buffer (0.1 % SDS) at 100 mV for 120 min. Electroblotting was performed onto polyvinylidene difluoride membranes (PVDF, Immun-Blot®, Bio-Rad) in Tris-Glycine buffer (0.3 % SDS, 20 % methanol) at 100 mV for 75 min (4 °C). The membranes were blocked in milk (5 %) in Tris-buffered saline supplemented with 0.1 vol% Tween20 (TBST) for 1 h. The membranes were incubated with the primary antibodies (in 2.5 % milk in TBST supplemented with 0.01 % NaN_3) overnight at 4 °C. After washing with TBST (3 · 5 min), the membranes were incubated with their corresponding secondary antibodies (in 2.5 % milk in TBST) for 45 min at room temperature. After washing with TBST (3 · 5 min) and Tris-buffered saline (5 min), proteins were visualized by use of the Clarity Western ECL Substrate (Bio-Rad).

D) Modulators and solutions for patch-clamping and calcium imaging

Experimental solutions were stored at -20 °C (for weeks) or +4 °C (for days). Glucose was added to the buffer on the experimental day. TRP-modulators were obtained from Sigma-Aldrich, diluted in DMSO, stored at -20 °C, and added to the reservoir with corresponding bath solution immediately before each experiment at a ratio of ≤ 1:1000.

<u>GSK1016790A</u> ($C_{28}H_{32}Cl_2N_4O_6S_2$): TRPV4 agonist
N-[(1S)-1-[[4-[(2S)-2-[[(2,4-Dichlorophenyl)sulfonyl]amino]-3-hydroxy-1-oxopropyl]-1-piperazinyl]carbonyl]-3-methylbutyl]benzo[b]thiophene-2-carboxamide

<u>GSK2193874</u> ($C_{37}H_{38}BrF_3N_4O$): TRPV4 antagonist
3-([1,4′-Bipiperidin]-1′-ylmethyl)-7-bromo-N-(1-phenylcyclopropyl)-2-[3-(trifluoromethyl)phenyl]-4-quinolinecarboxamide

<u>2-APB</u> ($C_{14}H_{16}BNO$): TRPV3 agonist
2-[(Diphenylboranyl)oxy]ethan-1-amine

Ion	concentrations (mmol · L^{-1})										
	I (NaCl)		II (NH_4Cl)			III (K-Gluconate)			IV (Butyrate$^-$)		
	Pip	Bath	Pip	Bath	Bath	Pip	Bath	Bath	Pip	Bath	Bath
	NaGlu	NaCl	NaGlu	NaCl	NH_4Cl	KGlu	NaCl	NaGlu	NaGlu	NaCl	NaBu
Na^+	135	135	135	135		6.8	138	138	140	140	140
NH_4^+					135						
K^+	5		5	5	5	127	5	5	5	5	5
Ca^{2+}		2		2	2	1.9	1.7	1.7		2	2
Mg^{2+}		2		2	2	2.3	0.9	0.9	0.9	1	1
Cs^+						5					
$NMDG^+$									3.2		
Cl^-	20	148	20	148	148	20.2	147	20.2	20	121	121
Gluconate$^-$	120		120			127		127	130	30	
Butyrate$^-$											30
HPO_4^-							1	1			
EGTA	5		5			10			5		
HEPES	10	10	10	10	10	10	10	10	1	1	1
Glucose		10		10	10		5	5		10	10
MgATP						1					
pH	7.4	7.4	7.4	7.4	7.4	7.2	7.4	7.4	7.4	7.4*)	7.4*)
osmolality§	305	305	315	315	315	315	315	315	316	316	316

§ in mosmol · kg^{-1} *) Solutions designated as “NaCl 6.4” and “NaBu 6.4” were buffered to pH 6.4 with MES.

E) Whole-cell patch-clamp experiments

Patch-clamp experiments were performed in a continuously perfused bath chamber at 23 °C [6,9,12,13]. A DMZ Universal Puller (Zeitz Instruments, Munich, Germany) was used to pull the pipettes. An EPC9 patch-clamp amplifier (HEKA Electronic, Lambrecht, Germany) recorded the currents using Patchmaster Software (HEKA Electronic). Agar bridges to ground the bath and for correction of the initial offset potential were made using the initial NaCl solution (Supplement, Part D). After seal formation and establishment of the whole-cell configuration, HEK-293 cells were clamped at a resting potential of -40 mV, from which the potential was stepped to values between +100 and -120 mV in 10 mV steps for 80 ms each at 5 kHz (Pulse protocol I), allowing analysis of current kinetics and determination of the reversal potential. Afterwards, a continuous pulse protocol in 20 mV steps with a low sampling rate (100 Hz) was applied to monitor solution changes (Pulse protocol II), alternating between these two pulse protocols automatically. The continuous pulse protocols were subsequently merged using Igor Pro 6.37 (WaveMetrics Inc., Lake Oswego, USA). After each overexpressing HEK-293 cell, a control cell was measured.

F) Data analysis

Data evaluation was performed using Igor Pro 6.37 (WaveMetrics Inc., Lake Oswego, USA). The Ussing chamber data were recorded at 10 points · min^{-1} and were averaged via binomial smoothing using Igor Pro Software before further analysis. The data analysis of patch-clamp experiments was essentially performed as described in Schrapers *et al.* [13]. Incomplete measurements or those with a series resistance ≤ 2 MOhm or an unstable current level were excluded. The reversal potentials (V_{rev}) were calculated by linear interpolation between the values above and below a current of zero in the corresponding IV-curve and corrected for liquid junction potentials using JPCalcWin software (School of Medical Sciences, Sydney, Australia) [1].
The relative permeability ratio $p(K^+)/p(Na^+)$ was estimated from the reversal potentials measured in cells filled with KGlu and superfused with NaGlu solution (Supplement, Part D, III) using the Goldman-Hodgkin-Katz equation [7,13]:

$$V_{rev} = \frac{R \cdot T}{F} \cdot ln\left(\frac{p(Na^+) \cdot [Na^+]_{out} + p(K^+) \cdot [K^+]_{out} + p(Cl^-) \cdot [Cl^-]_{in}}{p(Na^+) \cdot [Na^+]_{in} + p(K^+) \cdot [K^+]_{in} + p(Cl^-) \cdot [Cl^-]_{out}}\right)$$
$$\approx \frac{R \cdot T}{F} \cdot ln\left(\frac{p(Na^+) \cdot [Na^+]_{out}}{p(K^+) \cdot [K^+]_{in}}\right)$$

From this, it follows that:

$$p(K^+)/p(Na^+) = \frac{[Na^+]_{out}}{[K^+]_{in}} \cdot e^{-(V_{rev} \cdot \frac{F}{R \cdot T})}$$

Here, T is the temperature (296.15 K), F the Faraday constant (~ 96485 C · mol^{-1}), and R the universal gas constant (~ 8.3 J · K^{-1} · mol^{-1}). Note that the concentrations of $[Na^+]_{in}$ and $[K^+]_{out}$ were small (6.8 and 5 mmol · L^{-1}, respectively). Furthermore, the positive reversal potential in NaCl solution suggests that $p(Cl^-)$ was low, while $[Cl^-]_{out} = [Cl^-]_{in}$ = 20 mmol · L^{-1}, so that the contribution of Cl^- could be neglected. The relative permeability ratio $p(NH_4^+)/p(Na^+)$ was calculated from the difference of V_{rev} in NaCl and NH_4Cl solution (Supplement, Part D, II) using the standard relationship [7]:

$$V_{rev}\,(NaCl) - V_{rev}(NH_4Cl) \approx -\frac{R \cdot T}{F} \cdot ln\left(\frac{p(Na^+) \cdot [Na^+]_{out}}{p(NH_4^+) \cdot [NH_4^+]_{out}}\right)$$

G) Alignment of the human and bovine homologues of TRPV3 and TRPV4

Bovine TRPV4: QXI66840.1 (98.25 kDa)
Human TRPV4: NP_067638.3 (98.25 kDa)
Human TRPV4: NP_001170902.1 (95.01 kDa)
Human TRPV3: NP_001245134.1 (90.72 kDa)
Bovine TRPV3: ASO66785.1 (91.61 kDa)

Values e in parenthesis represent molecular weights of proteins, calculated using https://www.sciencegateway.org/tools/proteinmw.htm.

The regions of interest are shown in colour in the protein alignment and indicated by abbreviations as seen in the left column of the overview below. Corresponding investigations to these regions are shown in the right columns.

H^+	Activation by intracell. protons	(Gao, Yang et al. 2016) (Cao, Yang et al. 2012)	[5,3]
H^+	Activation by intracell. protons	(Wang, Yang et al. 2021)	[17]
2APB	Activation by 2-APB	(Hu, Grandl et al. 2009)	[8]
GSK	Activation by GSK1016790A	(Botte, Ulrich et al. 2020)	[2]
GSK*	Weak activation by GSK1016790A	(Botte, Ulrich et al. 2020)	[2]
pore	Pore region	(Voets, Prenen et al. 2002)	[16]
&gly	Gycolisation site	(Shikano, Ueda et al. 2011)	[14]
ATP	Sensitization by ATP	(Phelps, Wang et al. 2010)	[11]
ARD	Ankyrin repeat domain	(Phelps, Wang et al. 2010)	[11]
	Block by intracell. Ca^{2+} #)	(Nilius, Vriens et al. 2004) $[Ca^{2+}]_i$ > 400 nmol · L^{-1}	[10]
Ca^{2+}_e	Block by extracell. Ca^{2+}	(Watanabe, Vriens et al. 2003)	[18]
Ca^{2+}_i	Activation by intracell. Ca^{2+}	(Nilius, Vriens et al. 2004) $[Ca^{2+}]_i$ < 400 nmol · L^{-1}	[10]

#) location unclear

```
bV4: QXI66840.1      MADPGEGPRVGPGEMAESPGDESGPPGG-----------EAFPLSSLANLFEGEDG-SPS     48
hV4: NP_067638.3     MADSSEGPRAGPGEVAELPGDESGTPGG-----------EAFPLSSLANLFEGEDG-SLS     48
hV4: NP_001170902.1  MADSSEGPRAGPGEVAELPGDESGTP----------------------------------     26
hV3: NP_001245134.1  -------MKAHPKEMVPLMGKRVAAPSGNPAILPEKRPAEITPTKKSAHFFLEIEGFEPN     53
bV3: ASO66785.1       MSLCRTAMKAHPKEMVPLTGRRATIPFVNPAIMQEKRPSEITPTKKSAHFFLEIEGFEPN     60
                             :. * *:.   * .   *

QXI66840.1          PSPADGGRSTGPGDGRPNLRMKFQGAFRKGVPNPIDLLESTLYESSVVPGPKKAPMDSLF      108
NP_067638.3         PSPADASRPAGPGDGRPNLRMKFQGAFRKGVPNPIDLLESTLYESSVVPGPKKAPMDSLF      108
NP_001170902.1      ------------GDGRPNLRMKFQGAFRKGVPNPIDLLESTLYESSVVPGPKKAPMDSLF      74
NP_001245134.1      PTVAKTS--------PPVFSKPMDSNIRQCISGNCDDMDSP-----------QSPQ----      90
ASO66785.1          PTVAKTS--------PPIFSKPMDSNIRQCVSGNCDDMDSP-----------QSPQ----      97
                                    * :   ::. :*: : .  * ::*            ::*

QXI66840.1          DYGTYRHHPSDNKRWRRKVIEKQPQSPKAPAPQPPP----ILKVFNRPILFDIVSRGSTA      164
NP_067638.3         DYGTYRHHSSDNKRWRKKIIEKQPQSPKAPAPQPPP----ILKVFNRPILFDIVSRGSTA      164
NP_001170902.1      DYGTYRHHSSDNKRWRKKIIEKQPQSPKAPAPQPPP----ILKVFNRPILFDIVSRGSTA      130
NP_001245134.1      -----------------DDVTETPSNPNSPSAQLAKEEQRRKKRRLKKRIFAAVSEGCVE      133
ASO66785.1          -----------------DDVTETPSNPNSPSANLAKEEQRRKKKRLKKRIFTAVSEGCVE      140
                                     . : : *..*::*: :         *   :  :*  **.*..         ARD

QXI66840.1          DLDGLLPFLLTHKKR--------LTDEEFREPSTGKTCLPKALLNLSNGRNDTIPVLLDI      216 ATP
NP_067638.3         DLDGLLPFLLTHKKR--------LTDEEFREPSTGKTCLPKALLNLSNGRNDTIPVLLDI      216 ATP
NP_001170902.1      DLDGLLPFLLTHKKR--------LTDEEFREPSTGKTCLPKALLNLSNGRNDTIPVLLDI      182 ATP
NP_001245134.1      ELVELLVELQELCRRRHDEDVPDFLMHKLTASDTGKTCLMKALLNINPNTKEIVRILLAF      193
ASO66785.1          ELLELLGELQELCKRRHSLDVPDFLMHKLTALDTGKTCLMKALLNINPNTKEIVRILLAF      200
                    :*  **  *    :*        :  .::   .****** *****:. . :: : :** :   ARD

QXI66840.1          AERTGNMREFINSPFRDIYYRGQTALHIAIERRCKHYVELLVAQGADVHAQARGRFFQPK      276
NP_067638.3         AERTGNMREFINSPFRDIYYRGQTALHIAIERRCKHYVELLVAQGADVHAQARGRFFQPK      276
NP_001170902.1      AERTGNMREFINSPFRDIYYRGQTALHIAIERRCKHYVELLVAQGADVHAQARGRFFQPK      242
NP_001245134.1      AEENDILGRFINAEYTEEAYEGQTALNIAIERRQGDIAALLIAAGADVNAHAKGAFFNPK      253
```

```
ASO66785.1          AEENDILDRFINAEYTEEAYEGQTALNIAIERRQGDITAALIAAGADVNAHAKGVFFNPK      260
                    **... : .***: : :  *.*****:******  . .   *:* ****:*:*:* **:**     ARD

QXI66840.1          DEGGYFYFGELPLSLAACTNQPHIVNYLTENPHKKADMRRQDSRGNTVLHALVAIADNTR      336
NP_067638.3         DEGGYFYFGELPLSLAACTNQPHIVNYLTENPHKKADMRRQDSRGNTVLHALVAIADNTR      336
NP_001170902.1      DEGGYFYFGELPLSLAACTNQPHIVNYLTENPHKKADMRRQDSRGNTVLHALVAIADNTR      302
NP_001245134.1      YQHEGFYFGETPLALAACTNQPEIVQLLMEHE--QTDITSRDSRGNNILHALVTVAEDFK      311
ASO66785.1          YQHEGFYFGETPLALAACTNQPEIVQMLMENE--QTDITSQDSRGNNILHALVTVAEDFK      318
                     :   ***** **:********.**: * *:   ::*:  :*****.:*****::*:: :      ARD

QXI66840.1          ENTKFVTKMYDLLLLKCARLFPDSNLEAVLNNDGLSPLMMAAKTGKIGIFQHIIRREVTD      396
NP_067638.3         ENTKFVTKMYDLLLLKCARLFPDSNLEAVLNNDGLSPLMMAAKTGKIGIFQHIIRREVTD      396
NP_001170902.1      ENTKFVTKMYDLLLLKCARLFPDSNLEAVLNNDGLSPLMMAAKTGKIGIFQHIIRREVTD      362
NP_001245134.1      TQNDFVKRMYDMILLRSGN----WELETTRNNDGLTPLQLAAKMGKAEILKYILSREIKE      367
ASO66785.1          TQNDFVKRMYDMILLRSRT----WELETTRNNDGLTPLQLAAKMGKAEILKYILSREIKD      374
                     :..**.:***::**:.         :**:. *****:** :*** **  *:::*: **:.:   ARD

QXI66840.1          EDTRHLSRKFKDWAYGPVYSSLYDLSSLDTCGEETSVLEILVYNSKIENRHEMLAVEPIN      456
NP_067638.3         EDTRHLSRKFKDWAYGPVYSSLYDLSSLDTCGEEASVLEILVYNSKIENRHEMLAVEPIN      456
NP_001170902.1      EDTRHLSRKFKDWAYGPVYSSLYDLSSLDTCGEEASVLEILVYNSKIENRHEMLAVEPIN      422
NP_001245134.1      KRLRSLSRKFTDWAYGPVSSSLYDLTNVDTTT-DNSVLEITVYNTNIDNRHEMLTLEPLH      426 2APB
ASO66785.1          KRLRSLSRKFTDWAYGPVSSSLYDLTNVDTTT-DNSVLEIIVYNTNIDNRHEMLTLEPLH      433 2APB
                    :  * *****.******* ******:.:**   : ***** ***::*:******::**::

QXI66840.1          ELLRDKWRKFGAVSFYINVVSYLCAMVIFTLTAYYQPLEGT---PPYPYRTTMDYLRLAG      513 GSK
NP_067638.3         ELLRDKWRKFGAVSFYINVVSYLCAMVIFTLTAYYQPLEGT---PPYPYRTTVDYLRLAG      513 GSK
NP_001170902.1      ELLRDKWRKFGAVSFYINVVSYLCAMVIFTLTAYYQPLEGT---PPYPYRTTVDYLRLAG      479 GSK
NP_001245134.1      TLLHMKWKKFAKHMFFLSFCFYFFYNITLTLVSYYRPREEEAIPHPLALTHKMGWLQLLG      486
ASO66785.1          TLLHMKWKKFAKYMFFLSFCFYFFYNITLTLVSYYRPREEEALPHPLALTHKMGWLQLLG      493
                     **: **:**.   *::..  *:   : :**.:**:* *       *     .:.:*:* *

QXI66840.1          EIITLFFTGILFFFFTNIKDLFMKKCPGVNSLFIDGSFQLLYFIYSVLVIVSAALYLAGIEA      573
NP_067638.3         EVITLFFTGVLFFFFTNIKDLFMKKCPGVNSLFIDGSFQLLYFIYSVLVIVSAALYLAGIEA      573
NP_001170902.1      EVITLFFTGVLFFFFTNIKDLFMKKCPGVNSLFIDGSFQLLYFIYSVLVIVSAALYLAGIEA      539
NP_001245134.1      RMFVLIWAMCISVKEGIAIFLLRPSDLQSILSDAWFHFVFFIQAVLVILSVFLYLFAYKE      546 H+
ASO66785.1          RMFVLIWAMFISVKEGIAIFLLRPSDLQSILSDAWFHFVFFAQAVLVILSVFLYLFAYKE      553 H+
                    .::.*: .: : ..:   :*: :  .::*:: *. *::::*  :****:*. *** . :

QXI66840.1          YLAVMVFALVLGWMNALYFTRGLKLTGTYSIMIQKILFKDLFRFLLVYLLFMIGYASALV      633 GSK
NP_067638.3         YLAVMVFALVLGWMNALYFTRGLKLTGTYSIMIQKILFKDLFRFLLVYLLFMIGYASALV      633 GSK
NP_001170902.1      YLAVMVFALVLGWMNALYFTRGLKLTGTYSIMIQKILFKDLFRFLLVYLLFMIGYASALV      599 GSK*
NP_001245134.1      YLACLVLAMALGWANMLYYTRGFQSMGMYSVMIQKVILHDVLKFLFVYIVFLLGFGVALA      606
ASO66785.1          YLACLVLAMALGWANMLYYTRGFQSMGMYSVMIQKVILHDVLKFLFVYIVFLLGFGVALA      613
                    *** :*:*:.*** * **:***::  * **:****:::*::**:**::*::*:. **.

QXI66840.1          SLLNPCANLKVCDEDHANCTVPTYPSCRDSETFSTFLLDLFKLTIGMGDLEMLSSTKYPV      693 pore
NP_067638.3         SLLNPCANMKVCNEDQTNCTVPTYPSCRDSETFSTFLLDLFKLTIGMGDLEMLSSTKYPV      693 &gly
NP_001170902.1      SLLNPCANMKVCNEDQTNCTVPTYPSCRDSETFSTFLLDLFKLTIGMGDLEMLSSTKYPV      659 Ca2+e
NP_001245134.1      SLIEKCPK------DNK--------DCSSYGSFSDAVLELFKLTIGLGDLNIQQNSKYPI      652 pore
ASO66785.1          SLIEKCPK------SHE--------NCSSYGSFSDAVLELFKLTIGLGDLNIQQNSKYPI      659 pore
                    **:: * :      .:         .* .  :**  :*:*******:***::  ..:***:

QXI66840.1          VFIILLVTYIILTFVLLLNMLIALMGETVGQVSKESKHIWKLQWATTILDIERSFPVFLR      753
NP_067638.3         VFIILLVTYIILTFVLLLNMLIALMGETVGQVSKESKHIWKLQWATTILDIERSFPVFLR      753
NP_001170902.1      VFIILLVTYIILTFVLLLNMLIALMGETVGQVSKESKHIWKLQWATTILDIERSFPVFLR      719
NP_001245134.1      LFLFLLITYVILTFVLLLNMLIALMGETVENVSKESERIWRLQRARTILEFEKMLPEWLR      712 H+ &
ASO66785.1          LFLFLLITYVILTFVLLLNMLIALMGETVENVSKESERIWRLQRARTILEFEKILPEWLR      719 2APB
                    :*::**:**:******************* :*****::**:** * ***::*: :* :**

QXI66840.1          KAFRSGEMVTVGKSSDGTPDRRWCFRVDEVNWSHWNQNLGIINEDPGKNENYQYYGFSHT      813
NP_067638.3         KAFRSGEMVTVGKSSDGTPDRRWCFRVDEVNWSHWNQNLGIINEDPGKNETYQYYGFSHT      813
NP_001170902.1      KAFRSGEMVTVGKSSDGTPDRRWCFRVDEVNWSHWNQNLGIINEDPGKNETYQYYGFSHT      779
NP_001245134.1      SRFRMGELCKVAE-----DDFRLCLRINEVKWTEWKTHVSFLNEDPGPVRRTADFNKIQD      767 H+
ASO66785.1          SRFRMGELCKVAE-----DDFRLCLRINEVKWTEWKTHVSFLNEDPGPGRRTADSNKIQD      774 H+
                    . ** **: .*.:      * * *:*::**:*:.*: ::.::*****  .     .   :

QXI66840.1          VGRLRRDRWSSVVPRVVELNKNSNPDEVVVPLDNVGNPSCDGHQQSYPPKWRTDDAPL      871 Ca2+i
NP_067638.3         VGRLRRDRWSSVVPRVVELNKNSNPDEVVVPLDSMGNPRCDGHQQGYPRKWRTDDAPL      871 Ca2+i
NP_001170902.1      VGRLRRDRWSSVVPRVVELNKNSNPDEVVVPLDSMGNPRCDGHQQGYPRKWRTDDAPL      837 Ca2+i
NP_001245134.1      SSRNNSKTTLNAFEEVEEF-----PETSV----------------------------      791
ASO66785.1          SSRSNSKTTLNAFEEIDEF-----PETSV----------------------------      798
                     .* . .   ... .: *:      *:  *
```

References

1. Barry PH, Lynch JW (1991) Liquid junction potentials and small cell effects in patch-clamp analysis. J Membr Biol 121:101-117. doi:10.1007/BF01870526
2. Botte M, Ulrich AKC, Adaixo R, Gnutt D, Brockmann A, Bucher D, Chami M, Bocquet N, Ebbinghaus-Kintscher U, Puetter V, Becker A, Egner U, Stahlberg H, Hennig M, Holton SJ (2020) Cryo-EM structural studies of the agonist complexed human TRPV4 ion-channel reveals novel structural rearrangements resulting in an open-conformation. bioRxiv:2020.2010.2013.334797. doi:10.1101/2020.10.13.334797
3. Cao X, Yang F, Zheng J, Wang K (2012) Intracellular proton-mediated activation of TRPV3 channels accounts for the exfoliation effect of α-hydroxyl acids on keratinocytes. J Biol Chem 287:25905-25916. doi:10.1074/jbc.M112.364869
4. Gálfi P, Gäbel G, Martens H (1993) Influences of extracellular matrix components on the growth and differentiation of ruminal epithelial cells in primary culture. Res Vet Sci 54:102-109. doi:10.1016/0034-5288(93)90018-b
5. Gao L, Yang P, Qin P, Lu Y, Li X, Tian Q, Li Y, Xie C, Tian JB, Zhang C, Tian C, Zhu MX, Yao J (2016) Selective potentiation of 2-APB-induced activation of TRPV1-3 channels by acid. Sci Rep 6:20791. doi:10.1038/srep20791
6. Georgi MI, Rosendahl J, Ernst F, Gunzel D, Aschenbach JR, Martens H, Stumpff F (2014) Epithelia of the ovine and bovine forestomach express basolateral maxi-anion channels permeable to the anions of short-chain fatty acids. Pflugers Archiv : European journal of physiology 466:1689-1712. doi:10.1007/s00424-013-1386-x
7. Hille B (2001) Ion Channels of Excitable Membranes. 3rd edn. Sinauer Associates, Sunderland, Mass. doi:10.4236/jbm.2020.82005
8. Hu H, Grandl J, Bandell M, Petrus M, Patapoutian A (2009) Two amino acid residues determine 2-APB sensitivity of the ion channels TRPV3 and TRPV4. Proc Natl Acad Sci U S A 106:1626-1631. doi:10.1073/pnas.0812209106
9. Liebe F, Liebe H, Kaessmeyer S, Sponder G, Stumpff F (2020) The TRPV3 channel of the bovine rumen: localization and functional characterization of a protein relevant for ruminal ammonia transport. Pflugers Archiv : European journal of physiology 472:693-710. doi:10.1007/s00424-020-02393-2
10. Nilius B, Vriens J, Prenen J, Droogmans G, Voets T (2004) TRPV4 calcium entry channel: a paradigm for gating diversity. Am J Physiol Cell Physiol 286:C195-205. doi:10.1152/ajpcell.00365.2003
11. Phelps CB, Wang RR, Choo SS, Gaudet R (2010) Differential regulation of TRPV1, TRPV3, and TRPV4 sensitivity through a conserved binding site on the ankyrin repeat domain. J Biol Chem 285:731-740. doi:10.1074/jbc.M109.052548
12. Rosendahl J, Braun HS, Schrapers KT, Martens H, Stumpff F (2016) Evidence for the functional involvement of members of the TRP channel family in the uptake of Na+ and NH4 + by the ruminal epithelium. Pflügers Archiv - European Journal of Physiology 468:1333-1352. doi:10.1007/s00424-016-1835-4
13. Schrapers KT, Sponder G, Liebe F, Liebe H, Stumpff F (2018) The bovine TRPV3 as a pathway for the uptake of Na+, Ca2+, and NH4+. PloS one 13:e0193519. doi:10.1371/journal.pone.0193519
14. Shikano M, Ueda T, Kamiya T, Ishida Y, Yamada T, Mizushima T, Shimura T, Mizoshita T, Tanida S, Kataoka H, Shimada S, Ugawa S, Joh T (2011) Acid inhibits TRPV4-mediated Ca(2)(+) influx in mouse esophageal epithelial cells. Neurogastroenterol Motil 23:1020-1028, e1497. doi:10.1111/j.1365-2982.2011.01767.x
15. Stumpff F, Georgi MI, Mundhenk L, Rabbani I, Fromm M, Martens H, Günzel D (2011) Sheep rumen and omasum primary cultures and source epithelia: barrier function aligns with expression of tight junction proteins. J Exp Biol 214:2871-2882. doi:10.1242/jeb.055582
16. Voets T, Prenen J, Vriens J, Watanabe H, Janssens A, Wissenbach U, Bödding M, Droogmans G, Nilius B (2002) Molecular determinants of permeation through the cation channel TRPV4. J Biol Chem 277:33704-33710. doi:10.1074/jbc.M204828200
17. Wang H, Yang P, Lu Y, Wang J, Jeon J, Wang Q, Tian JB, Zang B, Yu Y, Zhu MX (2021) Mechanisms of proton inhibition and sensitization of the cation channel TRPV3. J Gen Physiol 153. doi:10.1085/jgp.202012663
18. Watanabe H, Vriens J, Janssens A, Wondergem R, Droogmans G, Nilius B (2003) Modulation of TRPV4 gating by intra- and extracellular Ca2+. Cell Calcium 33:489-495. doi:10.1016/s0143-4160(03)00064-2
